# Total Synthesis of Phorbazole B

**DOI:** 10.3390/molecules25204848

**Published:** 2020-10-21

**Authors:** Yngve Guttormsen, Magnus E. Fairhurst, Sunil K. Pandey, Johan Isaksson, Bengt Erik Haug, Annette Bayer

**Affiliations:** 1 Department of Chemistry, UiT The Arctic University of Norway, Hansine Hansens veg 54, 9037 Tromsø, Norway; yngve.guttormsen@uit.no (Y.G.); johan.isaksson@uit.no (J.I.); 2 Department of Chemistry and Centre for Pharmacy, University of Bergen, Allégaten 41, 5007 Bergen, Norway; magnus.fairhurst@uib.no (M.E.F.); sunil.pandey@uib.no (S.K.P.)

**Keywords:** phorbazole, oxazole, pyrrole, late-stage chlorination

## Abstract

Phorbazoles are polychlorinated heterocyclic secondary metabolites isolated from a marine sponge and several of these natural products have shown inhibitory activity against cancer cells. In this work, a synthesis of the trichlorinated phorbazole B using late stage electrophilic chlorination was developed. The synthesis relied on the use of an oxazole precursor, which was protected with an iodine in the reactive 4-position, followed by complete chlorination of all pyrrole positions. Attempts to prepare phorbazole A and C, which contain a 3,4-dichlorinated pyrrole, were unsuccessful as the desired chlorination pattern on the pyrrole could not be obtained. The identities of the dichlorinated intermediates and products were determined using NMR techniques including NOESY/ROESY, 1,1-ADEQUATE and high-resolution CLIP-HSQMBC.

## 1. Introduction

**Figure 1:**
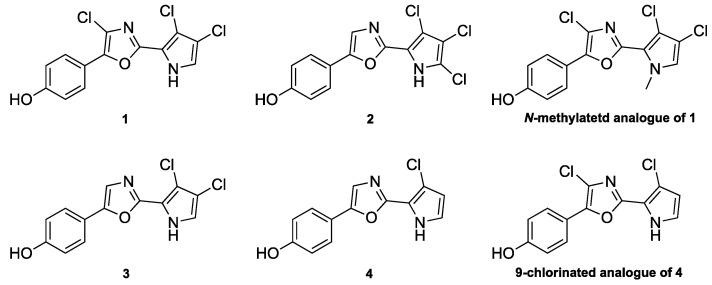
Phorbazoles A–D (**1**–**4**).

Phorbazoles A–D (**1**–**4**, Figure 1) are polychlorinated heterocyclic secondary metabolites isolated from the marine sponge *Phorbas aff. Clathrata* off the coast of South Africa in 1994 [1].

The phorbazoles were the first natural compounds isolated with the 2-(2′-pyrrolyl)oxazole fragment. They were also the first examples of chlorinated pyrroles found in nature. Later on, a *N*-methylated analogue of **1** and the 9-chlorinated analogue of **4** were isolated from the marine mollusk *Aldisa andersoni* [2]. No bioactivity has so far been reported for **2**–**4**, while **1** and its *N*-methylated analogue and the 9-chlorinated analogue of **4** have been found to have a feeding deterrence effect, which suggests that they are involved in chemical defense [2]. The two latter compounds have also been found to inhibit growth of cancer cells in vitro [2]. While the phorbazoles are biosynthetically produced by cyclization of dipeptides of tyrosine and proline, a synthesis of **3** reported in 2001 relied on a Robinson–Gabriel cyclodehydration of a precursor carrying the appropriately chlorinated pyrrole [3].

**Figure 2:**
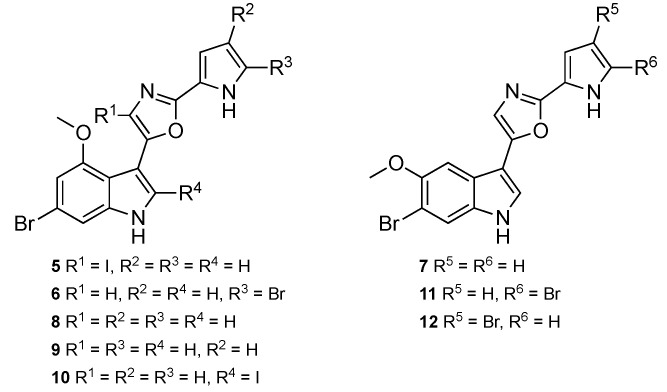
Breitfussins A–H (**5**–**12**).

Breitfussin A–H (**5**–**12**, Figure 2) constitute the second class of marine natural products containing the pyrrole-oxazole motif [4,5]. Like the phorbazoles, the breitfussins are highly halogenated natural products derived from dipeptide precursors, but with tryptophan instead of tyrosine.

We have earlier reported on the total syntheses of **5**–**8**, which were based on the utilization of two palladium-catalyzed cross couplings to install the indole and pyrrole onto the oxazole core, followed by halogenation of the pyrrole at a late stage in the case of **6** [5,6]. An alternative synthesis of **6** (and by coincidence also **8**) based on application of the Robinson–Gabriel cyclodehydration to form the oxazole ring combined with a late stage bromination strategy [7], has also been reported.

In this work, we report the total synthesis of **2** via late-stage chlorination and attempts at synthesizing **1** and **3** using the same strategy.

## 2. Results and Discussion

### 2.1. Retrosynthetic Analysis of **2**

**Scheme 1:**
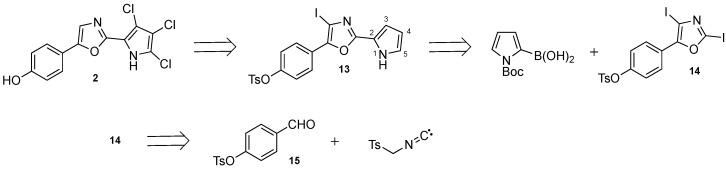
Retrosynthetic analysis for **2**.

We envisioned that the phorbazoles should be accessible via chlorination at a late stage, and a retrosynthetic analysis of **2**, which has not been prepared before, is shown in Scheme 1.

Due to the intrinsic propensity of both the oxazole and the pyrrole to undergo electrophilic aromatic substitution, the regioselectivity is difficult to control. The Chen group nicely illustrated the subtle inherent reactivity differences in the synthesis of **6**, where changing the solvent from acetone to tetrahydrofuran (THF)/pyridine altered the regioselectivity of the halogenation from the oxazole 4-position to the pyrrole 5-position [7]. Our strategy is based on our realization from the breitfussin syntheses that the iodine in the oxazole 4-position is acid-labile and may act as a protecting group also toward synthesizing phorbazoles. We thus envisioned that **2** could be obtained by chlorination of compound **13**, where the oxazole 4-position is protected. Access to **13** was planned through Suzuki Miyaura coupling on diiodide **14**, which we planned to obtain from the Van Leusen methyl isocyanide synthon [8] after cyclisation to the oxazole and subsequent deiodination as we have reported for the breitfussin syntheses [5,6].

### 2.2. Synthesis of ***2***

**Scheme 2:**
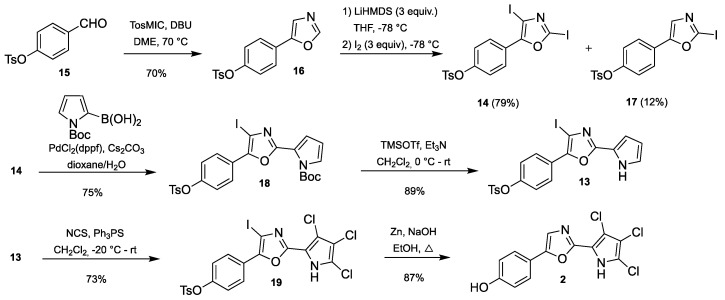
Synthesis of **2** by late-stage chlorination.

The synthesis of the common intermediate **13** commenced with a modified Van Leusen oxazole synthesis on the previously reported aldehyde **15** (Scheme 2) [9].

Protection of the phenol is required both for the lithiation step and to avoid chlorination on the phenol later in the synthesis [10]. In order to avoid deprotection of the tosyl group, 1,8-diazabicyclo [5.4.0] undec-7-en (DBU) was used as base instead of K_2_CO_3_. Heating the aldehyde **15** and the TosMIC reagent with DBU in 1,2-dimethoxyethane (DME) (70 °C, 5 h) gave oxazole **16** in 70% yield. This material was dilithiated by treatment with lithium bis(hexamethylsilyl)amide (LiHMDS) in THF at −78 °C for 1 h before addition of iodine to give the diiodinated oxazole **14** in 79% yield. While this protocol also resulted in the formation of the monoiodinated 2-iodo oxazole **17** in 12% yield, it does not require prolonged reaction times as has been reported for other methods [11]. The diiodo oxazole **14** was subsequently coupled to Boc-protected (1*H*-pyrrol-2-yl)boronic acid in a Suzuki-Miyaura reaction. The coupling reaction proceeded with excellent regioselectivity favoring the 2-substituted oxazolyl-pyrrole **18** in 75% yield after 68 h at rt. We were not able to detect any product stemming from coupling at the oxazole 4-position, although this result cannot be completely ruled out. Next, the Boc group was removed using trimethylsilyl trifluoromethanesulfonate (TMSOTf) under basic conditions to give the common intermediate **13** in high yield (89%). Chlorination of the common intermediate **13** using Palau’chlor [12], gave a mixture of di- and trichlorinated products, while the recently developed procedure of Gustafson [13] using *N*-chlorosuccinimide (NCS)/Ph_3_PS resulted in complete conversion. At first 3.07 equiv. of NCS and 20 mol% of Ph_3_PS was added; however, when HRMS analysis revealed uncomplete reaction (see experimental part for details), a further 0.2 equiv. of NCS was added. Thus, using a total of 3.27 equiv. of NCS, we obtained the desired trichlorinated pyrrole **19** in 73% yield. No chlorination on the phenyl ring was observed. Next, we opted to deiodinate **19** using the conditions identified through our total syntheses of breifussins A–D (**5**–**9**, Figure 2); however, treatment of **19** under acidic conditions did not give any traces of deiodinated product. On the other hand, simultaneous deprotection of the tosyl and iodine protecting groups of **19** was achieved using zinc in refluxing ethanolic sodium hydroxide, to give **2** in 87% yield. The NMR spectra of synthetic material in (CD_3_)_2_SO (see Figures S1 and S2) matched the reported spectra of the natural phorbazole B [1] (see Table S1 for a comparison).

### 2.3. Attempted Synthesis of ***1*** and ***3***

**Scheme 3:**
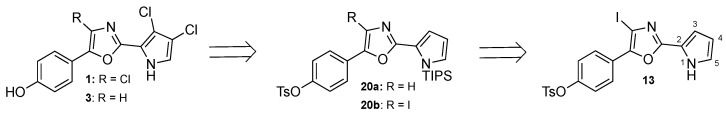
Retrosynthetic analysis for **1** and **3**.

We envisioned that it might be possible to access natural products **1** and **3** by shielding the pyrrole 5-position with a bulky triisopropylsilyl (TIPS) group on the pyrrole *N*-atom, giving **20a** and **20b** as intermediates for the synthesis of **1** and **3**, respectively (Scheme 3). The literature suggests that the extent of shielding that TIPS protection of the pyrrole can infer on C-5 (our numbering, see Scheme 3) of the pyrrole, varies depending on the chlorination reagent and solvent that is employed; however, conditions that result in full shielding of the 2-position of pyrrole itself have not been achieved [14]. On the other hand, for bromination and iodination, the TIPS group is reported to offer excellent shielding as both exposure of *N*-TIPS-protected pyrrole to NBS or NIS resulted in clean formation of the 3-bromo or 3-iodo derivative [14,15,16,17].

**Scheme 4:**
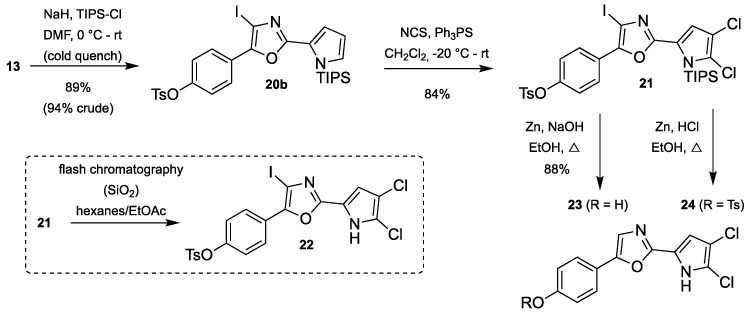
Attempted synthesis of **3**.

The TIPS protection of **13** (Scheme 4) turned out to be a delicate step, as a reaction using THF as solvent was successful with 96% yield the first time it was tested; however, all later attempts using this solvent only gave back the starting material. The use of DMF as a solvent and NaH as a base [18], and quenching the reaction by addition of crushed ice consistently gave the crude product of **20b** in around 94% yield and with high purity. Unfortunately, **20b** proved to be somewhat unstable upon flash chromatography purification and was therefore, for the most part, used in the next step without purification. Upon prolonged storage, the TIPS group was cleaved off to give back the starting material.

Chlorination of **20b** was performed analogously to the chlorination of the unprotected pyrrole in **13** (see above). By using 2.05 equivalents of NCS, one major dichlorinated product was formed as evident by ^1^H-NMR analysis of the crude product. The clean conversion of **20b** to one major dichlorinated product was very encouraging; however, upon global deprotection (vide infra), the NMR spectra of the material obtained did not match those reported for natural product **3** [1]. It turned out that, instead of the desired chlorination of the pyrrole 3- and 4-positions, selective dichlorination on the pyrrole 4- and 5-positions had occurred to give regioisomer **21** in an 84% isolated yield (see NMR discussion below for the structure assignment of **21**).

Upon flash column purification of the crude product following the chlorination reaction, we experienced that the material decomposed when ethyl acetate (0–20%) in hexanes was used as the eluent, to give TIPS-deprotected **22** as the major product. We speculate that the presence of acetic acid in the ethyl acetate might provide an explanation for the observed decomposition. By using CH_2_Cl_2_ (0–100%) in heptane as the eluent, decomposition was avoided completely.

Complete deprotection—i.e., removal of the iodine, the tosyl and the TIPS protecting groups—could be facilitated using Zn in refluxing ethanolic NaOH (vide supra) to give **23** in high yield (88%). Under acidic conditions, using Zn in refluxing ethanolic HCl, the tosyl group was retained providing compound **24**—the tosyl-protected analogue of **23**. In the face of the undesired regioselectivity for the dichlorination step and the lability of the TIPS group of **20b**, further attempts to synthesize **1** and **3** were suspended.

The unambiguous determination of the chlorination pattern of fully protected **21** and partially deprotected **24** was not trivial because of the absence of protons in the area. The spectral assignments were made by a combination of ^1^H-1D, ^13^C-1D, HSQC, HMBC, COSY, NOESY/ROESY, 1,1-ADEQUATE and high-resolution CLIP-HSQMBC (see Figures S36–S48). In order to establish the chlorination pattern, it was necessary to unambiguously determine the position of the pyrrole CH-proton.

From the spectral data obtained of **24**, we could exclude that this compound contained a proton on the pyrrole 5-position due to the absence of any NOESY or ROESY correlation to the pyrrole NH proton (Figure S48). The experimental chemical shift for the pyrrole CH was determined to be 111.3 ppm, which is not consistent with this carbon atom being next to a nitrogen, for which a chemical shift of around 130 ppm would have been expected. Thus, in order to differentiate between positions 3 and 4 on the pyrrole, we had to establish which carbon atoms carried chlorine and differentiate these from C-2 of the pyrrole, which experimentally resonates at 117.3 ppm—inconveniently near the two chlorinated carbon atoms C-5 (117.1 ppm) and C-4 (111.9 ppm). The pyrrole C-4 signal was sharp and a ~0.6 Hz ^35,37^Cl isotope shift could be directly observed at 214 MHz field for ^13^C. The pyrrole C-5 resonance was however broad and the isotope shift could not be observed directly in ^13^C-NMR nor indirectly in a high-resolution selective CLIP-HSQMBC.^1^ A weak long-range ^n^*J*_CH_ was observed between the proton and the carbon in the 2-position of the oxazole, and supports the proton sitting in position 3 of the pyrrole, but since the coupling was very weak it could not be used alone to rule out a potential four-bond coupling from the pyrrole 4 position.

In intermediate **21**, the ^35,37^Cl isotope shift could however be observed for both the C-4 (113.8 ppm) and the C-5 (123.4 ppm) of the pyrrole, in both ^13^C-NMR and selective CLIP-HSQMBC (Figure S41), presumably because the absence of the exchangeable pyrrole NH no longer causes line broadening of the neighboring carbon resonances. The presence of the *N*-TIPS group in **21** did, however, affect the chemical shifts significantly. On the other hand, the long range coupling constants between the oxazole C-2 and the pyrrole H-3 (2.0 Hz), the pyrrole C-2 and the pyrrole H-3 (5.4 Hz) and the pyrrole C-5 and H-3 (9.5 Hz) were not significantly changed by the TIPS group, and the measurements of these couplings for all intermediates were used to ensure that the chemical shift assignment of the chlorinated pyrrole carbons could be transferred from **21** to **24**.^2^ Knowing the unambiguous assignments of pyrrole carbons C-2 and C-5, 1,1-ADEQUATE correlations from pyrrole H-3 to C-2 and C-4 could be used to conclusively determine the chlorination pattern.

## 3. Materials and Methods

### 3.1. General Information

Chemicals and solvents were purchased from Sigma-Aldrich and used as delivered unless otherwise stated. All moisture sensitive reactions were carried out under argon atmosphere in oven-dried (130 °C) equipment that has been cooled down under vacuum. Anhydrous THF was either obtained from a sodium/benzophenone still or an anhydrous solvent delivery system (SPS-800 system from M. Braun GmbH, Garching, Germany). Flash column chromatography was performed using silica gel from Merck (Silica gel 60, 0.040–0.063 mm). Thin layer chromatography (TLC) analyses were performed on aluminum sheets coated with Merck TLC silica gel 60 F254 and visualization was achieved by using ultraviolet light (254 nm) or a solution of sodium permanganate. The NMR experiments were recorded on a Bruker 400 MHz, a Bruker BioSpin AV500 or a Bruker BioSpin Ascend spectrometer operating at 850 MHz for ^1^H equipped with an inverse-detected triple resonance (TCI) cryoprobe. ^1^H and ^13^C chemical shifts (δ) are reported in ppm with reference to the solvent residual peak (CDCl_3_: δH 7.26 and δC 77.16; (CD_3_)_2_SO: δH 2.50 and δC 39.98). All coupling constants are given in Hz. Positive and negative ion electrospray ionization mass spectrometry was conducted on a Thermo electron LTQ Orbitrap XL spectrometer (Thermos, Bremen, Germany) with methanol as solvent. Flash chromatography was performed on a Biotage SP1 HPFC system (Biotage, Uppsala, Sweden) or a PuriFlash XS 420 system (Interchim, Montlucon Cedex, France).

### 3.2. Methods

#### 3.2.1. Synthesis of Phorbazole B (**2**)

The trichlorinated pyrrole **19** (7.65 mg, 12.5 µmol) was dissolved in ethanol (0.2 mL) and 6 M aqueous NaOH (50 µL) was added. The reaction mixture was heated to reflux, zinc (12 mg, 190 µmol) was added and heating was continued for 1 h. To the reaction mixture 1 mL 10% NaHCO_3_ was added and extracted with 3 × 1 mL CHCl_3_ on a Biotage phase separator with a Na_2_SO_4_ filter plug. The solution was evaporated to give the title compound.

Colorless solid; yield 3.58 mg (87%); ^1^H-NMR (400 MHz, (CD_3_)_2_SO) δ = 13.57 (s, 1H), 9.86 (s, 1H), 7.63 (d, *J* = 7.7, 2H), 7.60 (s, 1H),6.89 (d, *J* = 7.7, 2H); ^13^C-NMR (101 MHz, (CD_3_)_2_SO) δ = 158.0, 151.6, 150.3, 125.7, 121.1, 118.3, 115.9, 115.8, 115.0, 110.0, 108.5; HRMS (ESI) *m*/*z* calcd. for C_13_H_6_O_2_N_2_^35^Cl_3_ [M − H]^−^: 326.9500; found: 326.9496. The NMR spectra in (CD_3_)_2_SO (Table S1) matched the original spectra [1].

#### 3.2.2. Synthesis of 4-(4-Iodo-2-(1H-pyrrol-2-yl)oxazol-5-yl)phenyl 4-methyl-benzenesulfonate (**13**)

To a solution of Boc-protected pyrrole **18** (530 mg, 0.87 mmol) in anhydrous CH_2_Cl_2_ (5 mL) at 0 °C was slowly added Et_3_N (1.20 mL, 8.74 mmol) and TMSOTf (1.58 mL, 8.74 mmol). The reaction mixture was stirred at room temperature for 16 h, after which no starting material could be observed by TLC analysis. The reaction mixture was diluted with ethyl acetate, quenched with cold water and extracted with ethyl acetate (3 × 20 mL). The organic phase was concentrated and dissolved in a small amount of CH_2_Cl_2_ before adsorption on a Biotage snaplet precolumn and purified by flash chromatography on a Biotage SNAP Ultra column using an eluent with 0–80% ethyl acetate in heptane to give the title compound. 

Orange solid; yield 395 mg (89%); mp 53.4–54.7 °C; ^1^H-NMR (400 MHz, CDCl_3_) δ = 9.58 (bs, 1H), 7.93 (d, *J* = 8.8, 2H), 7.74 (d, *J* = 8.2, 2H), 7.34 (d, *J* = 8.0, 2H), 7.08 (d, *J* = 8.8, 2H), 7.00–6.96 (m, 1H), 6.91–6.87 (m, 1H), 6.34–6.30 (m, 1H), 2.46 (s, 3H); ^13^C-NMR (101 MHz, CDCl_3_) δ = 157.5, 149.6, 147.4, 145.7, 132.3, 130.0, 128.7, 127.0, 126.4, 122.9, 122.6, 119.1, 111.7, 110.8, 79.7, 29.9 (grease), 21.9: HRMS (ESI) *m*/*z* calcd. for C_20_H_16_O_4_N_2_IS [M + H]^+^: 506.9870; found: 506.9865.

#### 3.2.3. Synthesis of 4-(2,4-diiodooxazol-5-yl)phenyl 4-methylbenzenesulfonate (**14**)

Oxazole **16** (5.00 g, 15.9 mmol) was dissolved in anhydrous THF (75 mL) and cooled to −78 °C. Freshly prepared LiHMDS (1M in THF/hexanes, 47.6 mL, 47.6 mmol. Prepared by adding a 2.5 M *n*-BuLi solution in hexanes into HMDS in THF) was added dropwise. The resulting mixture was stirred at −78 °C for 1 h, during which the solution turned very viscous. A solution of iodine (12.10 g, 47.6 mmol) in anhydrous THF (10 mL) was added very slowly under thorough stirring. The resulting mixture was heated to room temperature for 1 h before 10% aqueous Na_2_S_2_O_3_ (100 mL) was added to quench the reaction and the resulting two layers were separated. The aqueous layer was extracted with ethyl acetate (2 × 50 mL) and the combined organic layers were washed with saturated NaCl (50 mL), dried over Na_2_SO_4_ and evaporated. The resulting residue was purified by flash chromatography on a Biotage SNAP Ultra column using an eluent with 5–40% ethyl acetate in heptane to give the title compound.

Yellow solid; yield 6.99 g (79%); mp 57.3–58.7 °C; ^1^H-NMR (400 MHz, CDCl_3_): δ = 7.86 (d, *J* = 8.9, 2H), 7.73 (d, *J* = 8.4, 2H), 7.33 (d, *J* = 8.0, 2H), 7.09 (d, *J* = 8.9, 2H), 2.46 (s, 3H); ^13^C-NMR (101 MHz, CDCl_3_): δ = 156.0, 150.3, 145.8, 132.3, 130.0, 128.7, 127.4, 125.3, 123.0, 101.4, 80.00, 21.9; HRMS (ESI) *m*/*z* calcd for C_16_H_11_O_4_KNI_2_S [M + K]^+^: 605.8130; found: 605.8129.

#### 3.2.4. Synthesis of 4-Formylphenyl 4-methylbenzenesulfonate (**15**)

4-Hydroxy-benzaldehyde (20.33 g, 166 mmol) and DMAP (68 mg, 0.61 mmol) were dissolved in a mixture of CH_2_Cl_2_ (200 mL) and Et_3_N (75 mL) and cooled to 0 °C. Tosyl chloride (31.7 g, 166 mmol) was dissolved in CH_2_Cl_2_ (60 mL) and added via an addition funnel for 20 min. The resulting mixture was left to heat to room temperature overnight and was then poured into water (200 mL). The phases were separated and the organic phase was washed with 1M aqueous HCl (2x100 mL), water (100 mL) and saturated NaCl (100 mL) before drying over MgSO_4_ and evaporation. The residue was dissolved in a small amount of ethyl acetate and heated to reflux before cooling to -20 °C overnight. The precipitated crystals were filtered off and dried to afford 25.90 g of the title compound. The mother liquid was evaporated and the crystallization was repeated to give an additional 2.81 g of the title compound. The remaining mother liquid was evaporated and crystallized from 10–15% water in ethanol to give an additional 9.10 g of the title compound.

Colorless solid; yield 37.81 g (82%); mp 72.5–73.5 °C; ^1^H-NMR (400 MHz, CDCl_3_): δ = 9.96 (s, 1H), 7.82 (d, *J* = 8.2, 2H), 7.71 (d, *J* = 7.9, 2H), 7.32 (d, *J* = 8.0, 2H), 7.16 (d, *J* = 8.2, 2H), 2.44 (s, 3H); ^13^C-NMR (101 MHz, CDCl_3_) δ = 190.7, 154.0, 146.0, 134.9, 132.1, 131.4, 130.1, 128.6, 123.2, 21.8; HRMS (ESI) *m*/*z* calcd for C_14_H_11_O_4_S [M − H]^−^: 375.0384; found: 375.0384.

#### 3.2.5. Synthesis of 4-(Oxazol-5-yl)phenyl 4-methylbenzenesulfonate (**16**)

Aldehyde **15** (2.0 g, 7.24 mmol) and TosMIC (1.48 g, 7.60 mmol) were dissolved in DME (10 mL). DBU (1.14 mL, 7.60 mmol) was added using a syringe and the resulting mixture was heated to reflux until TLC analysis showed that the starting material had been consumed (approx. 5 h). The reaction mixture was evaporated, re-dissolved in a small amount of acetone, adsorbed onto a Biotage snaplet precolumn and purified by flash chromatography on a Biotage SNAP Ultra column using an eluent with 15–100% ethyl acetate in heptane to give the title compound.

Pale yellow solid; yield 1.59 g (70%); mp 112.2–114.1 °C; ^1^H-NMR (400 MHz, CDCl_3_) δ = 7.90 (s, 1H), 7.72 (d, *J* = 8.3, 2H), 7.57 (d, *J* = 8.8, 2H), 7.32 (m, 3H), 7.05 (d, *J* = 8.8, 2H), 2.45 (s, 3H); ^13^C-NMR (101 MHz, CDCl_3_) δ = 150.9, 150.5, 149.6, 145.7, 132.3, 130.0, 128.7, 126.8, 125.8, 123.2, 122.2, 21.9; HRMS (ESI) *m*/*z* calcd for C_16_H_14_NO_4_S^+^ [M + H]^+^: 354.0197, found 354.0201.

#### 3.2.6. Synthesis of 4-(2-Iodooxazol-5-yl)phenyl 4-methylbenzenesulfonate (**17**)

The title compound was isolated as a side-product during the synthesis of **14**.

Yellow solid; yield 837 mg (12%); mp 39.1–41.4 °C; ^1^H-NMR (400 MHz, CDCl_3_): δ = 7.71 (d, *J* = 8.4, 2H), 7.51 (d, *J* = 8.8, 2H), 7.31 (d, *J* = 8.0, 2H), 7.24 (s, 1H), 7.04 (d, *J* = 8.8, 2H), 2.46 (s, 3H); ^13^C-NMR (101 MHz, CDCl_3_): δ = 152.7, 149.9, 146.8, 145.8, 132.3, 130.0, 128.7, 126.0, 125.5, 124.0, 123.4, 29.8 (grease), 21.9; HRMS (ESI) *m*/*z* calcd for C_16_H_13_O_4_NIS [M + H]^+^: 441.9604; found: 441.9604.

#### 3.2.7. Synthesis of t-Butyl 2-(4-iodo-5-(4-(tosyloxy)phenyl)oxazol-2-yl)-1H-pyrrole-1-carboxylate (**18**)

Diiodooxazol **16** (3.22 g, 5.77 mmol), Cs_2_CO_3_ (5.64 g, 17.3 mmol), (*N*-Boc-pyrrol-2-yl)boronic acid (1.46 g, 6.93 mmol) and PdCl_2_(dppf)·CH_2_Cl_2_ (235 mg, 0.289 mmol) were dissolved in a mixture of degassed dioxane (18 mL) and degassed water (6 mL). The solution was stirred at room temperature for 6 h under argon upon which TLC analysis showed some remaining starting material. Additional boronic acid (0.5 equiv.) and catalyst (5 mol%) were added and the resulting mixture was stirred for 42 h after which TLC still showed starting material left. Additional boronic acid (0.5 equiv.) and catalyst (5 mol%) were added and the mixture was stirred for 18 h, after which TLC showed no sign of starting material. The mixture was filtered through a plug of celite, diluted with ethyl acetate, washed with water (20 mL) and saturated NaCl (20 mL), dried over Na_2_SO_4_ and evaporated. The residue was purified by flash chromatography on a Biotage SNAP Ultra column using an eluent with 0–80% ethyl acetate in heptane to give the title compound.

Orange solid; yield 2.64 g (75%); mp 73.1–73.3 °C; ^1^H-NMR (400 MHz, CDCl_3_): δ = 7.94 (d, *J* = 8.8, 2H), 7.73 (d, *J* = 7.9, 2H), 7.44 (s, 1H), 7.32 (d, *J* = 8.1, 2H), 7.26 (s, 1H), 7.15–7.02 (m, 2H), 6.75 (s, 1H), 6.29 (d, *J* = 3.5, 1H), 2.45 (s, 3H), 1.44 (s, 9H); ^13^C-NMR (101 MHz, CDCl_3_): δ = 156.6, 149.8, 149.2, 148.2, 145.7, 132.4, 130.0, 128.7, 127.2, 126.4, 125.3, 122.9, 119.7, 119.7, 111.3, 85.1, 79.3, 27.8, 21.9; HRMS (ESI) *m*/*z* calcd for C_25_H_24_O_6_N_2_IS [M + H]^+^: 607.0394; found: 607.0394.

#### 3.2.8. Synthesis of 4-(4-Iodo-2-(3,4,5-trichloro-1H-pyrrol-2-yl)oxazol-5-yl)phenyl 4-methylbenzenesulfonate (**19**)

2-(4-Iodooxazole)pyrrole **13** (101 mg, 0.20 mmol) and triphenylphosphine sulfide (12 mg, 40 µmol) were dissolved in CH_2_Cl_2_ (0.8 mL) and cooled to −20 °C. NCS (82 mg, 0.61 mmol) was added in one portion and the reaction mixture was allowed to heat to room temperature for 10 min. A small aliquot was subjected to HRMS analysis, which revealed that the trichlorination was not complete. The reaction mixture was again cooled to −20 °C and additional NCS (5 mg, 37 µmol) was added. After heating to room temperature no traces of dichlorinated compound(s) could be observed, and the reaction mixture was directly applied onto a Biotage SNAP Ultra column and purified by flash chromatography using 40–100% CH_2_Cl_2_ in heptane to give the title compound. This material was used in the next step without further purification; however, ^1^H-NMR analysis revealed the presence of minor amounts of residual solvent/and or grease. A small sample was purified further by normal and C18 flash chromatography for characterization by ^1^H- and ^13^C-NMR analysis.

Pale orange solid; yield 88 mg (73%); mp 97.7–99.4 °C; ^1^H-NMR (400 MHz, (CD_3_)_2_SO) δ = 8.02 (d, *J* = 8.9, 2H), 7.78 (d, *J* = 8.2, 2H), 7.50 (d, *J* = 8.2, 2H), 7.24 (d, *J* = 8.9, 2H), 2.44 (s, 3H); ^13^C-NMR (101 MHz, CDCl_3_) δ = 154.7, 149.4, 147.4, 146.5, 131.7, 130.8, 128.8, 127.3, 126.4, 123.3, 117.1, 115.0, 111.8, 109.2, 83.6, 21.7; HRMS (ESI) *m*/*z* calcd. for C_20_H_11_O_4_N_2_S^35^Cl_2_^37^ClI [M − H]^−^: 608.8526; found: 608.8514.

#### 3.2.9. Synthesis of 4-(4-Iodo-2-(1-(triisopropylsilyl)-1H-pyrrol-2-yl)oxazol-5-yl)-phenyl 4-methylbenzenesulfonate (**20b**)

2-(4-Iodo-oxazole)pyrrole **13** (479 mg, 0.95 mmol) was dissolved in anhydrous DMF (20 mL) and the solution was cooled at 0 °C in an ice/water bath. NaH (60% in mineral oil, 92 mg, 2.28 mmol) was added slowly and the color of the solution changed from green to orange. The flask was flushed with argon and stirring was continued for 15 min before TIPS-Cl (0.25 mL, 1.14 mmol) was added dropwise upon which the reaction mixture changed to a peachy color. Stirring was continued for 15 min at 0 °C before the cooling bath was removed and stirring continued at room temperature for 2 h when TLC analysis showed complete conversion of the starting material (TLC sample quenched with ice and extracted with ethyl acetate before spotting). The reaction mixture was cooled on ice, diluted with ethyl acetate (20 mL) and quenched by slow addition of crushed ice. Phases were separated, and the aqueous phase was extracted with ethyl acetate (2 × 25 mL). The combined organic phases were washed with water (25 mL) and saturated NaCl (25 mL), dried over Na_2_SO_4_, filtered and evaporated under reduced pressure. The residue was purified by flash chromatography using a Biotage SNAP Ultra column using 0–20% ethyl acetate in heptane to give the title compound. For the most part, the crude product from this step was used in the next step without further purification.

Colorless oil; 558 mg (89%); ^1^H-NMR (400 MHz, CDCl_3_) δ = 7.94 (d, *J* = 8.8, 2H), 7.74 (d, *J* = 8.4, 2H), 7.33 (d, *J* = 8.1, 2H), 7.12–7.02 (m, 4H), 6.35 (t, *J* = 3.1, 1H), 2.46 (s, 3H), 1.86 (hept, *J* = 7.6, 3H), 1.13 (d, *J* = 7.6, 18H); ^13^C-NMR (101 MHz, CDCl_3_) δ = 158.0, 149.4, 147.0, 145.7, 132.4, 131.1, 130.0, 128.7, 126.9, 126.7, 124.4, 122.8, 117.6, 111.1, 79.3, 29.84 (impurity), 21.9, 18.4, 13.5; HRMS (ESI) *m*/*z* calcd. for C_29_H_35_O_4_N_2_SiSINa [M + Na]^+^: 685.1024; found: 685.1022.

#### 3.2.10. Synthesis of 4-(2-(4,5-Dichloro-1-(triisopropylsilyl)-1H-pyrrol-2-yl)-4-iodo-oxazol-5-yl)phenyl 4-methylbenzenesulfonate (**21**)

TIPS-protected pyrrole **20b** (100 mg, 0.161 mmol) and triphenylphosphine sulfide (10 mg, 32 µmol) were dissolved in CH_2_Cl_2_ (0.8 mL) and cooled to −20 °C. NCS (44 mg, 0.330 mmol) was added in one batch, and the reaction mixture was allowed to warm to room temperature for 10 min. A small aliquot was subjected to HRMS analysis, which showed that complete dichlorination had occurred. The reaction mixture was directly applied onto a Biotage SNAP Ultra column and purified using flash chromatography using 0–100% CH_2_Cl_2_ in heptane to give the title compound.

Colorless oil; yield 99 mg (84%); ^1^H-NMR (400 MHz, CDCl_3_) δ = 7.91 (d, *J* = 8.8, 2H), 7.74 (d, *J* = 8.1, 2H), 7.34 (d, *J* = 8.1, 2H), 7.09 (d, *J* = 8.8, 2H), 6.75 (s, 1H), 2.46 (s, 3H), 1.72 (hept, *J* = 7.5, 3H), 1.10 (d, *J* = 7.5, 18H); ^13^C-NMR (101 MHz, CDCl_3_) δ = 157.1, 149.9, 148.7, 145.8, 132.4, 130.0, 128.7, 127.2, 126.1, 123.8, 123.4, 123.0, 118.5, 113.8, 79.1, 29.9 (imp.), 21.9, 18.5, 13.7; HRMS (ESI) *m*/*z* calcd. for C_29_H_33_N_2_O_4_N_2_SiS^35^Cl_2_IK [M + K]^+^: 768.9984; found: 768.9987.

#### 3.2.11. Synthesis of 4-(2-(4,5-Dichloro-1H-pyrrol-2-yl)oxazol-5-yl)phenol (**23**)

TIPS-protected dichlorinated pyrrole **21** (48 mg, 66 µmol) was dissolved in ethanol (0.7 mL) at 70 °C and 20% aqueous NaOH (0.3 mL) was added, upon which the color changed from clear to orange. The mixture was heated to reflux and, after 5 min, zinc (64 mg, 0.984 mmol) was added and heating was continued for 45 min. The reaction mixture was filtered through a pad of Celite and partitioned between ethyl acetate (10 mL) and water (10 mL). The aqueous phase was extracted with ethyl acetate (2 × 10 mL) and the combined organic phases were washed with saturated NaCl, dried over Na_2_SO_4_ and evaporated. The residue was adsorbed onto a Biotage snaplet precolumn using CH_2_Cl_2_ and purified by flash chromatography using a Biotage SNAP Ultra column using 0–100% ethyl acetate in heptane to give the title compound.

Colorless solid; yield 17 mg (88%); mp 246 °C (dec.); ^1^H-NMR (400 MHz, (CD_3_)_2_SO) δ = 13.12 (s, 1H), 9.82 (s, 1H), 7.62 (d, J = 8.4, 2H), 7.51 (s, 1H), 6.94–6.75 (m, 3H); 13C-NMR (101 MHz, (CD3)2SO) δ = 157.8, 153.0, 150.1, 125.6, 121.1, 118.9, 118.5, 115.8, 114.7, 109.12, 109.11; HRMS (ESI) *m*/*z* calcd. for C_13_H_9_O_2_N_2_^35^Cl_2_ [M + H]^+^: 295.0036; found: 295.0037. 

## 4. Conclusions

In conclusion, we have achieved the total synthesis of phorbazole B in six steps with an overall yield of 23%, showing the utility of a simple catalyzed chlorination and demonstrating the use of iodine as a protection group for oxazoles. Furthermore, we have concluded that phorbazoles A and C were not accessible through our synthetic approach based on previous literature reports and our own observation of predominantly 4,5-dichlorination of the pyrrole instead of the desirable 3,4-dichlorination pattern of phorbazole A and C.

## Supplementary Materials

The following are available online, Figures S1–S22: 1D NMR spectra for **2** and **13**–**24**, Table S1: Comparison of spectral data for synthetic and isolated **2**, synthesis protocol for **24**, structural assignment of **21** and **24**, Figures S25–S37: 1D and 2D NMR spectra for assignment of chlorination pattern for **21** and **24**.
