# Supplementary material for: Total Synthesis of Phorbazole B
Source: Molecules. 2020 Oct 21;25(20):4848. doi: 10.3390/molecules25204848 (PMC7588006; doi:10.3390/molecules25204848)
Supplement: Supplementary file 1 [file molecules-25-04848-s001.pdf]

Supporting information for:

Total synthesis of phorbazole B

Yngve Guttormsen,[1] Magnus E. Fairhurst,[2] Sunil K. Pandey,[2] Johan Isaksson,[1]
Bengt Erik Haug,[2*] and Annette Bayer[1*]

1. Department of Chemistry, UiT The Arctic University of Norway, Hansine Hansens veg 54, 9037 Tromsø, Norway.
2. Department of Chemistry and Centre for Pharmacy, University of Bergen, Allégaten 41, 5020 Bergen, Norway.

Table of content:

Figure S1. 1H-NMR spectrum (400 MHz) of phorbazole B (**2**) 3

Figure S2. ^{13}C-NMR spectrum (101 MHz) of phorbazole B (**2**) 3

Figure S3. High resolution mass spectrum of phorbazole B (**2**) 4

Table S1. Comparison of the spectral data for synthetic and isolated phorbazole B (**2**) 5

Figure S4. ^{1}H-NMR spectrum (400 MHz) of **13** 6

Figure S5. 13C-NMR spectrum (101 MHz) of **13** 6

Figure S6. High resolution mass spectrum of **13** 7

Figure S7. ^{1}H-NMR spectrum (400 MHz) of **14** 7

Figure S8. ^{13}C-NMR spectrum (101 MHz) of **14** 8

Figure S9. High resolution mass spectrum of **14** 8

Figure S10. ^{1}H-NMR spectrum (400 MHz) of **15** 9

Figure S12. ^{1}H-NMR spectrum (400 MHz) of **16** 10

Figure S13. ^{13}C-NMR spectrum (101 MHz) of **16** 10

Figure S14.High resolution mass spectrum of **16.** 11

Figure S15. 1H-NMR spectrum (400 MHz) of **17** 11

Figure S16. ^{13}C-NMR spectrum (101 MHz) of **17** 12

Figure S17.High resolution mass spectrum of **17.** 12

Figure S18. ^{1}H-NMR spectrum (400 MHz) of **18** 13

Figure S19. ^{13}C-NMR spectrum (101 MHz) of **18** 13

Figure S20.High resolution mass spectrum of **18.** 14

Figure S21. 1H-NMR spectrum (400 MHz) of **19**. 14

Figure S22. 13C-NMR spectrum (101 MHz) of **19**. 15

Figure S23.High resolution mass spectrum of **19.** 15

Figure S24. ^{1}H-NMR spectrum (400 MHz) of **20b** 16

Figure S25. ^{13}C-NMR spectrum (101 MHz) of **20b**. 16

Figure S26. High resolution mass spectrum of **20b** 17

Figure S27. ^{1}H-NMR (400 MHz) spectrum of **21**. 17

Figure S28. ^{13}C-NMR (101 MHz) spectrum of **21**. 18

Figure S29. High resolution mass spectrum of **21**. 18

Figure S30. ^{1}H-NMR (850 MHz) spectrum of **22**. 19
Figure S31. ^{13}C-NMR (126 MHz) spectrum of **22**. 19
Figure S32. Low resolution MS of **22**. 20
Figure S33. ^{1}H-NMR (400 MHz) spectrum of **23**. 20
Figure S34. ^{13}C-NMR (101 MHz) spectrum of **23**. 21
Figure S35.High resolution mass spectrum of **23**. 21
Synthesis of 4-(2-(4,5-Dichloro-1H-pyrrol-2-yl)oxazol-5-yl)phenyl 4-methylbenzenesulfonate (**24**) 22
Figure S36. ^{1}H-NMR (400 MHz) spectrum of **24**. 22
Figure S35.High resolution mass spectrum of **24**. 23
Structural assignment for **21** and **24** 24
Figure S36. ^{1}H-NMR (850 MHz) spectrum of **21**. 24
Figure S37. ^{13}C-NMR (214 MHz) spectrum of **21.** 25
Figure S38. Superimposed HSQC and HMBC of **21**. 26
Figure S39. 1,1-ADEQUATE of **21**. 27
Figure S40. Selective CLIP-HSQMBC spectra of **21**. 28
Figure S41. Hires ^{13}C (top panel) and hires selective CLIP-HSQMBC f1 projection (bottom panel) of **21**. 29
Figure S42. ^{1}H-NMR (850 MHz) spectrum of **24**. 30
Figure S43. ^{13}C-NMR (214 MHz) spectrum of **24**. 31
Figure S44. Superimposed HSQC and HMBC of **24**. 32
Figure S45. 1,1-ADEQUATE of **24**. 33
Figure S46. Hires ^{13}C (top panel) and hires selective CLIP-HSQMBC f1 (bottom panel) projection of **24**. 34
Figure S47. Selective CLIP-HSQMBC of **24**. 35
Figure S48. NOESY and ROESY of **24**. 36
References 36

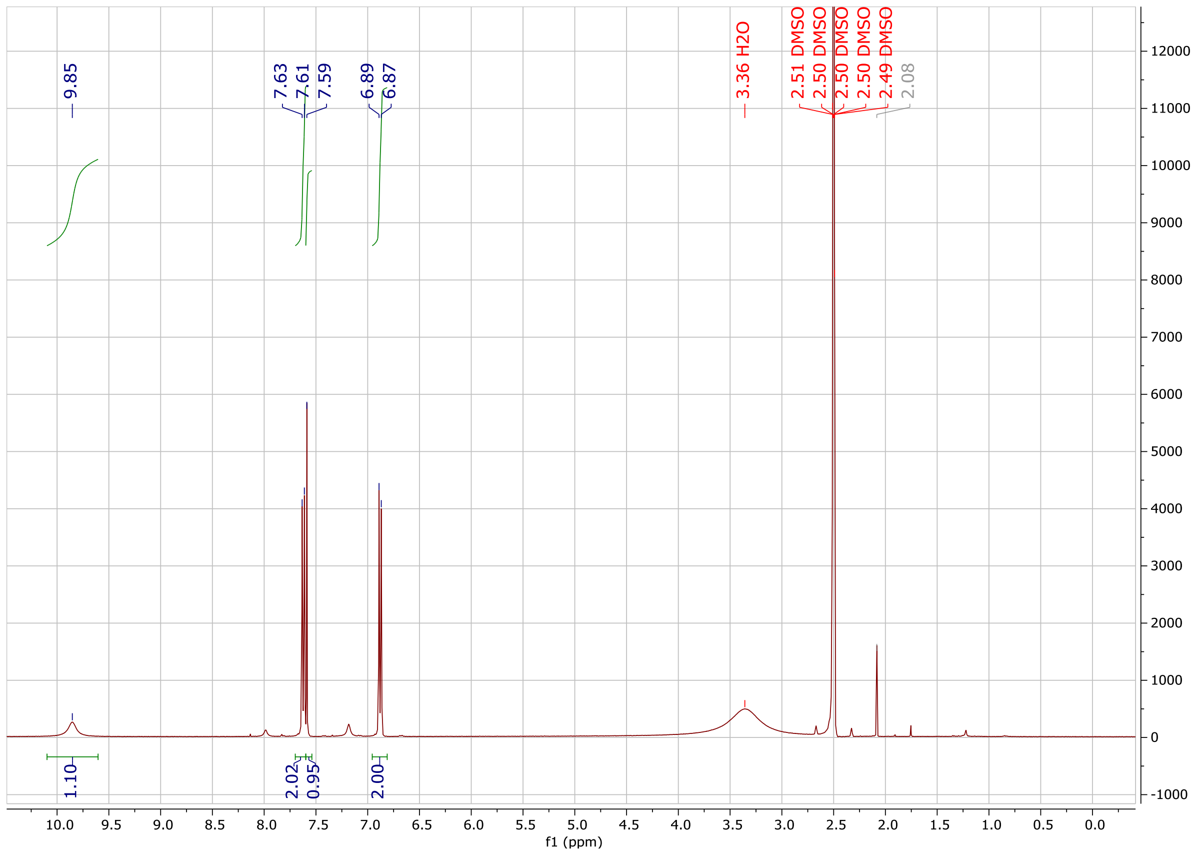

Figure S1. 1H-NMR spectrum (400 MHz) of phorbazole B (**2**)

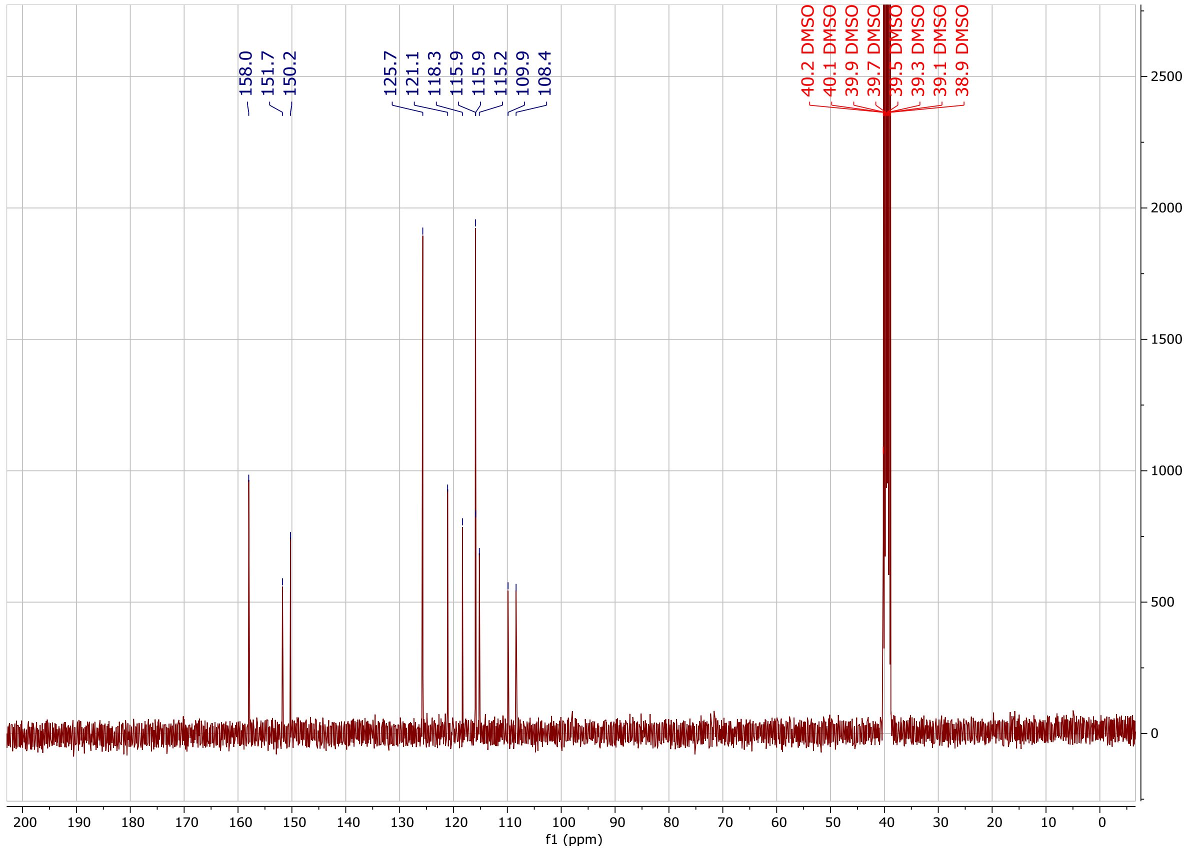

Figure S2. ^{13}C-NMR spectrum (101 MHz) of phorbazole B (**2**)

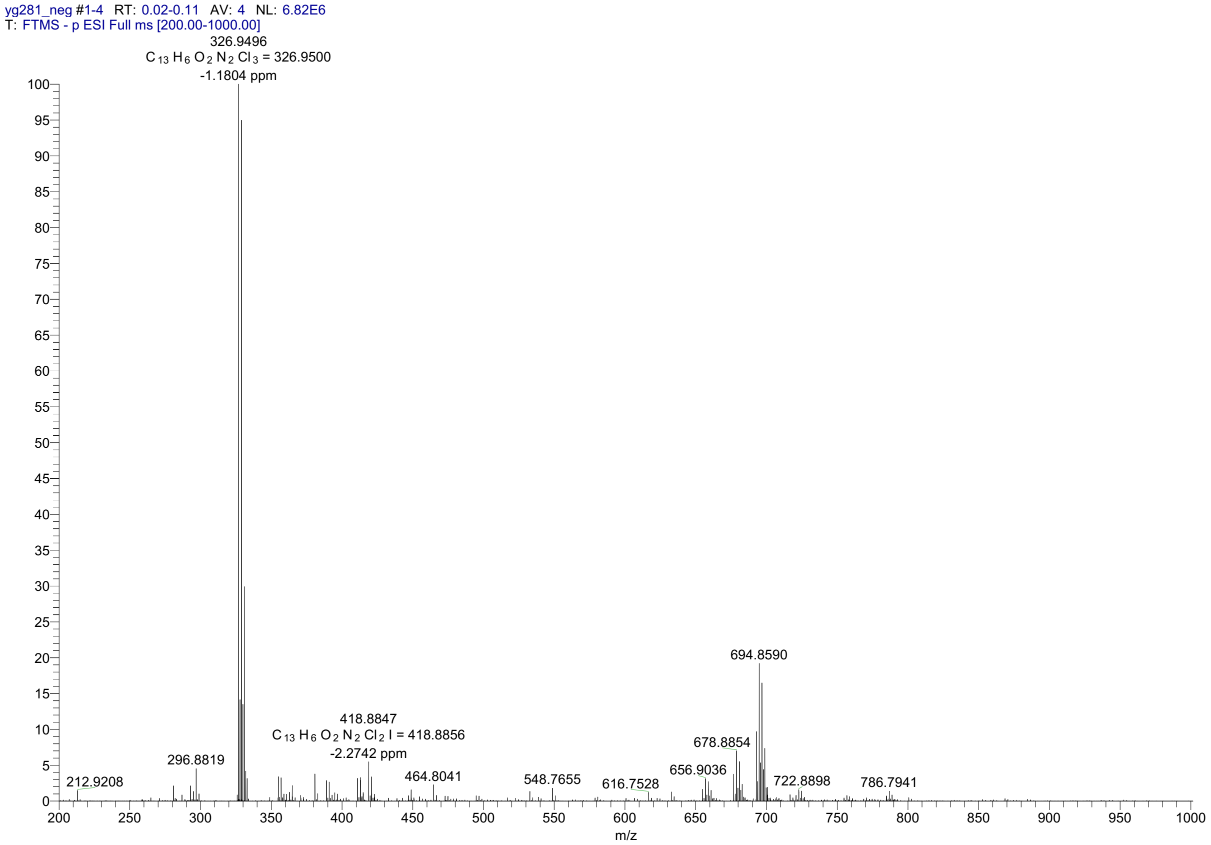

Figure S3. High resolution mass spectrum of phorbazole B (**2**)

Table S1. Comparison of the spectral data for synthetic and isolated phorbazole B (**2**)

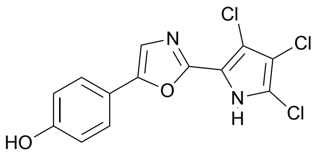

Synthetic **2**<br>^{1}H-NMR, 400 MHz, $(CD_3)_2SO$	Isolated **2**<br>^{1}H-NMR, 500 MHz, $(CD_3)_2SO$
13.57 (s, 1H)	-
9.85 (s, 1H)	-
7.62 (d, *J* = 7.7 Hz, 2H)	7.63 (d, *J* = 8.0 Hz)
7.59 (s, 1H)	7.60 (s)
6.88 (d, *J* = 7.7 Hz, 2H)	6.88 (d, *J* = 8.0 Hz)
Synthetic **2**<br>^{13}C-NMR, 101 MHz, $(CD_3)_2SO$	Isolated **2**<br>^{13}C-NMR, 150 MHz, $(CD_3)_2SO$
158.0	158.0
151.7	151.6
150.2	150.3
125.7	125.7
121.1	121.1
118.3	118.3
115.9	115.9
115.8	115.8
115.2	115.0
109.9	109.9
108.4	108.6

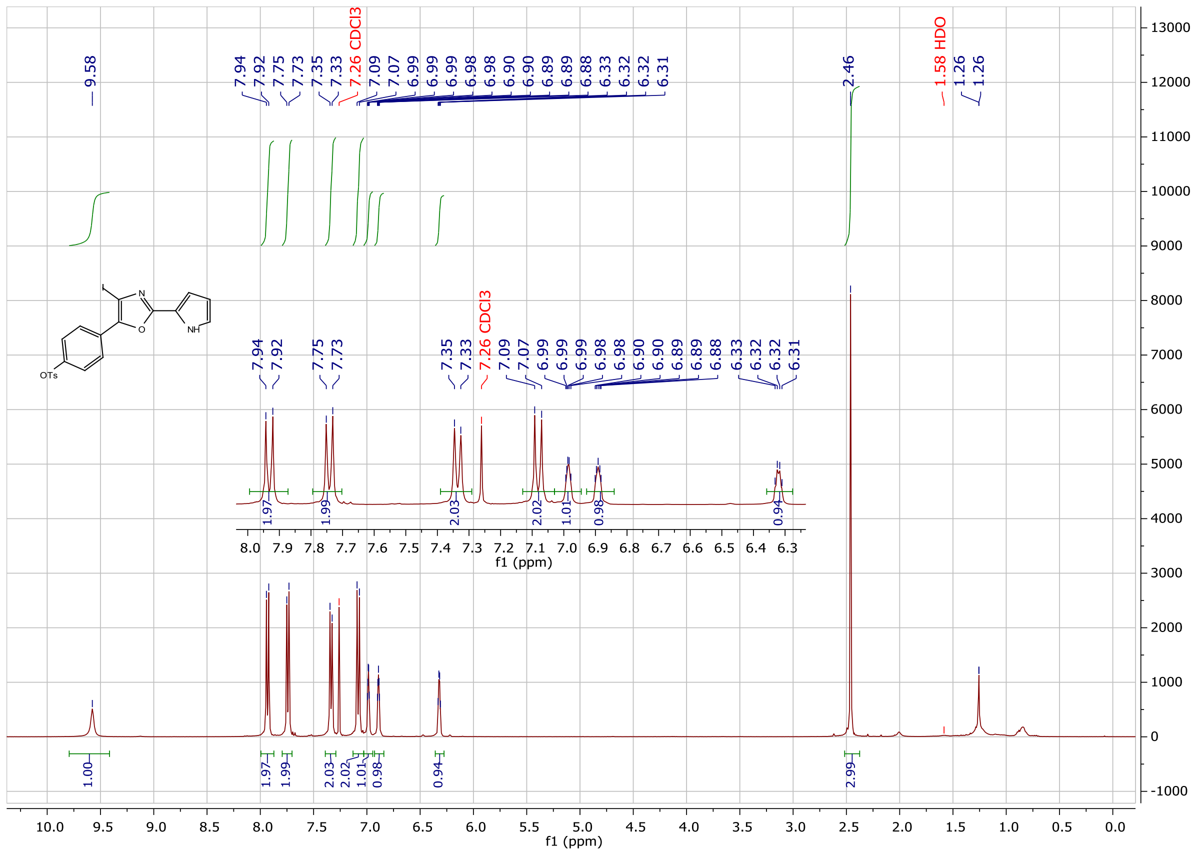

Figure S4. ^{1}H-NMR spectrum (400 MHz) of **13**

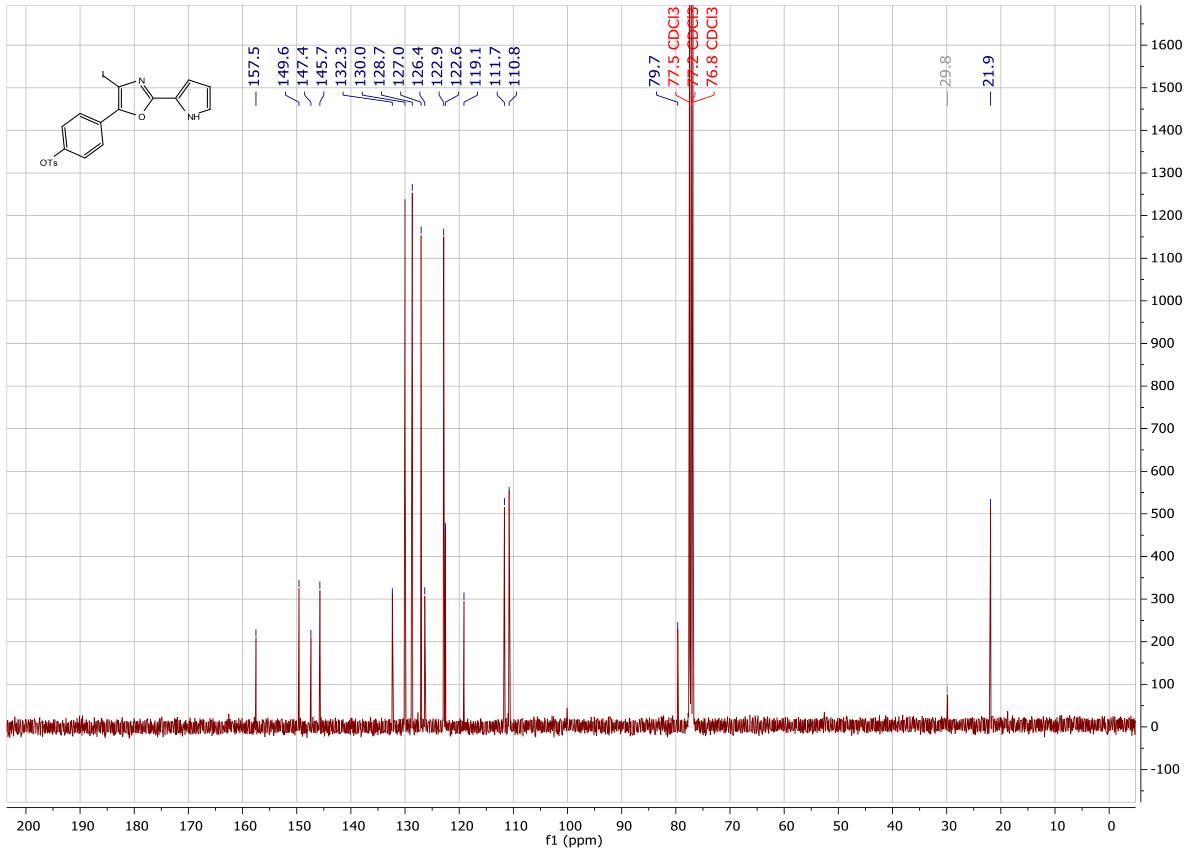

Figure S5. 13C-NMR spectrum (101 MHz) of **13**

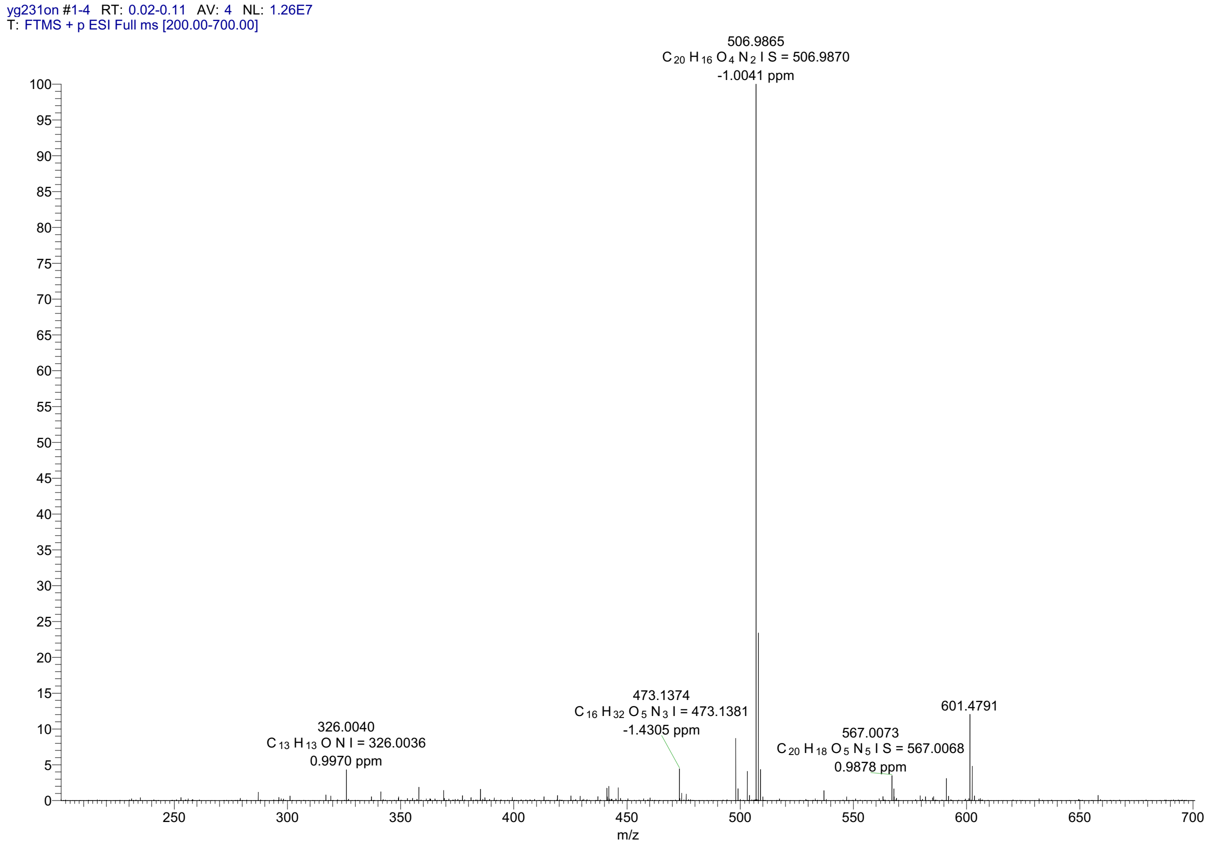

Figure S6. High resolution mass spectrum of **13**

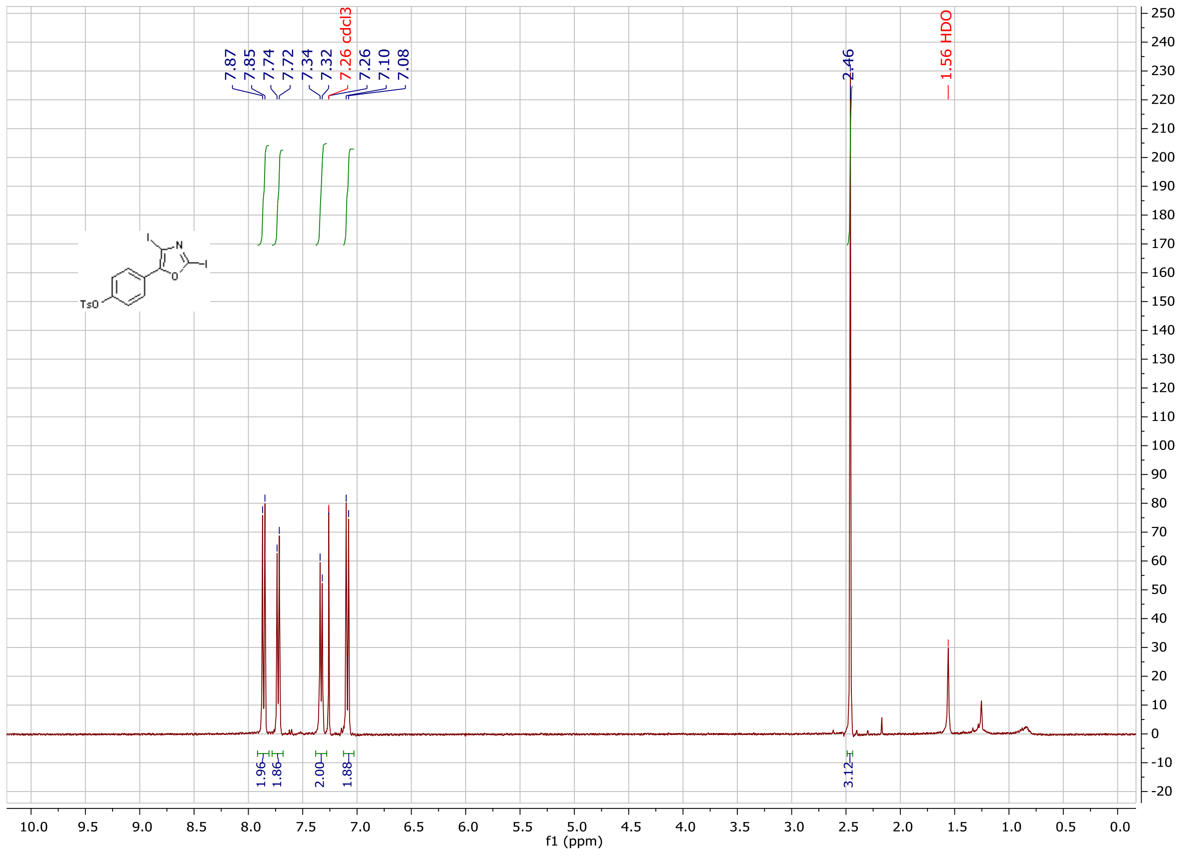

Figure S7. ^{1}H-NMR spectrum (400 MHz) of **14**

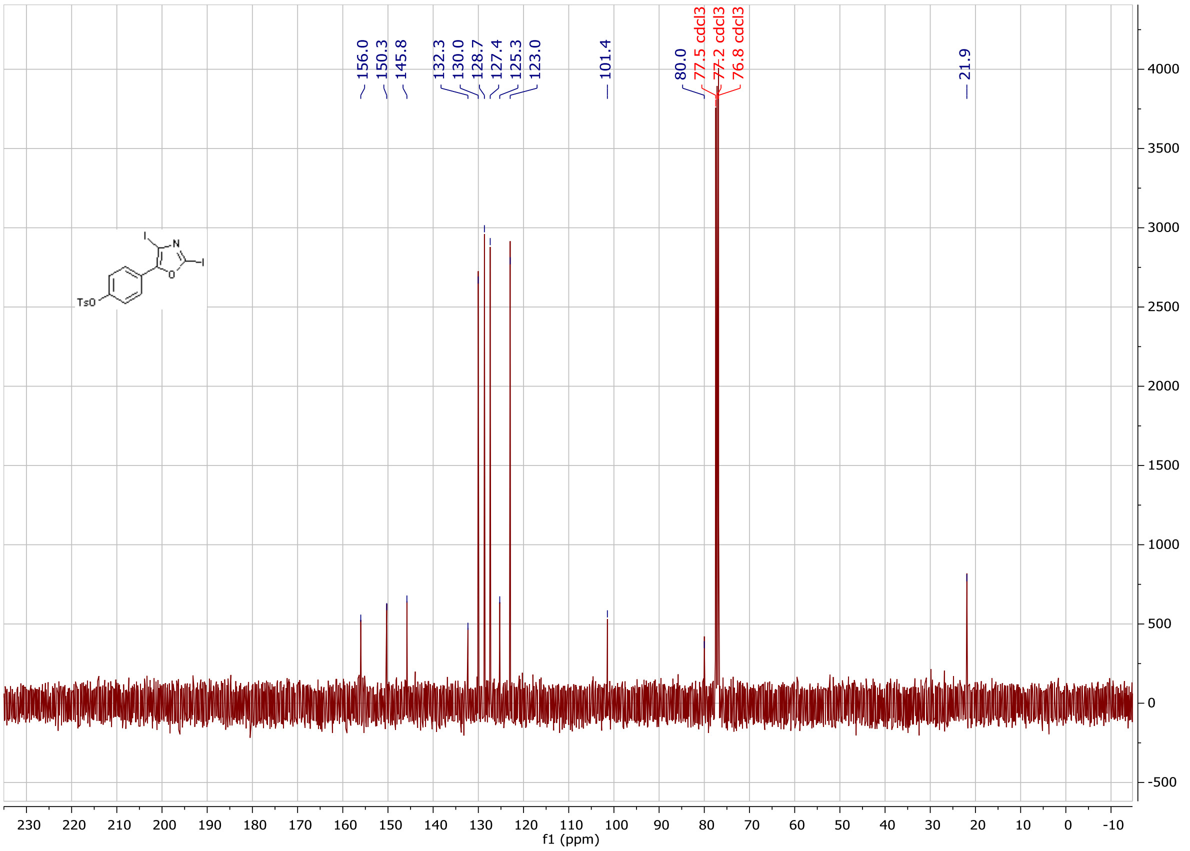

Figure S8. ^{13}C-NMR spectrum (101 MHz) of **14**

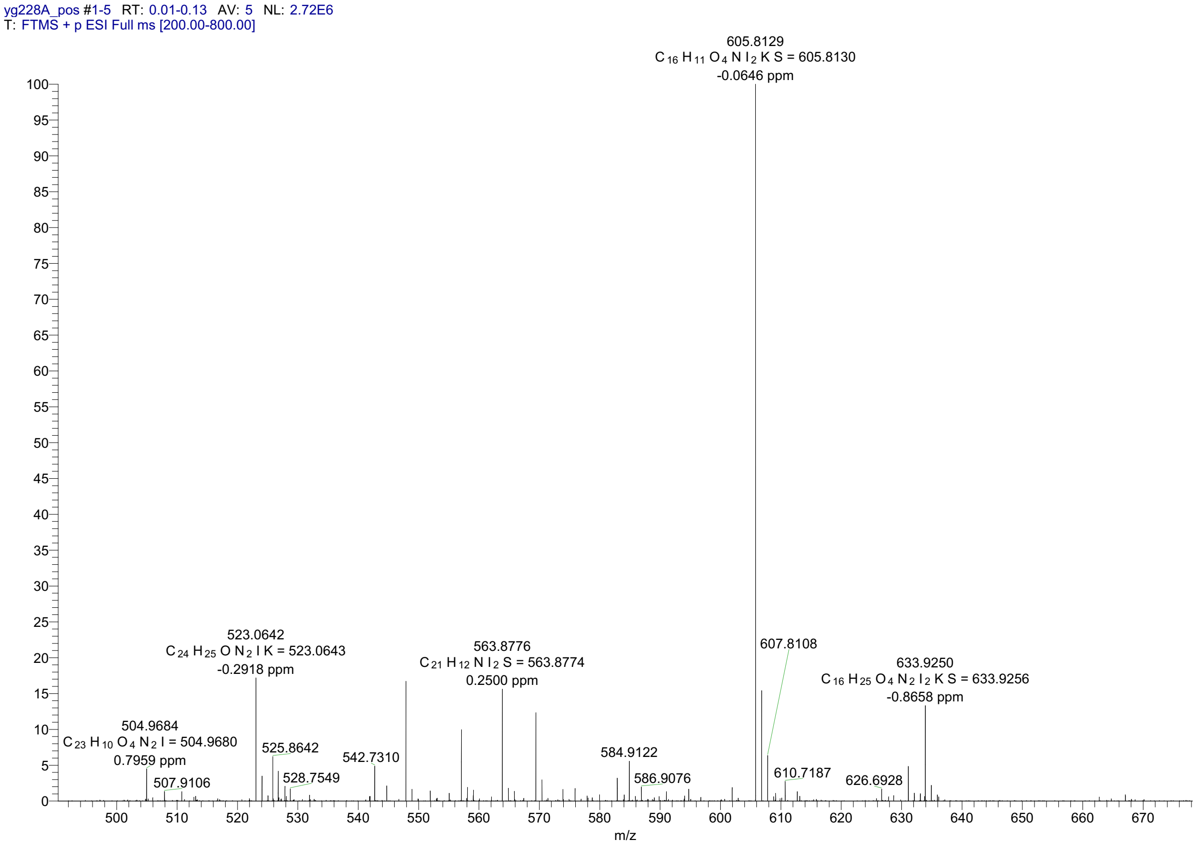

Figure S9. High resolution mass spectrum of **14**

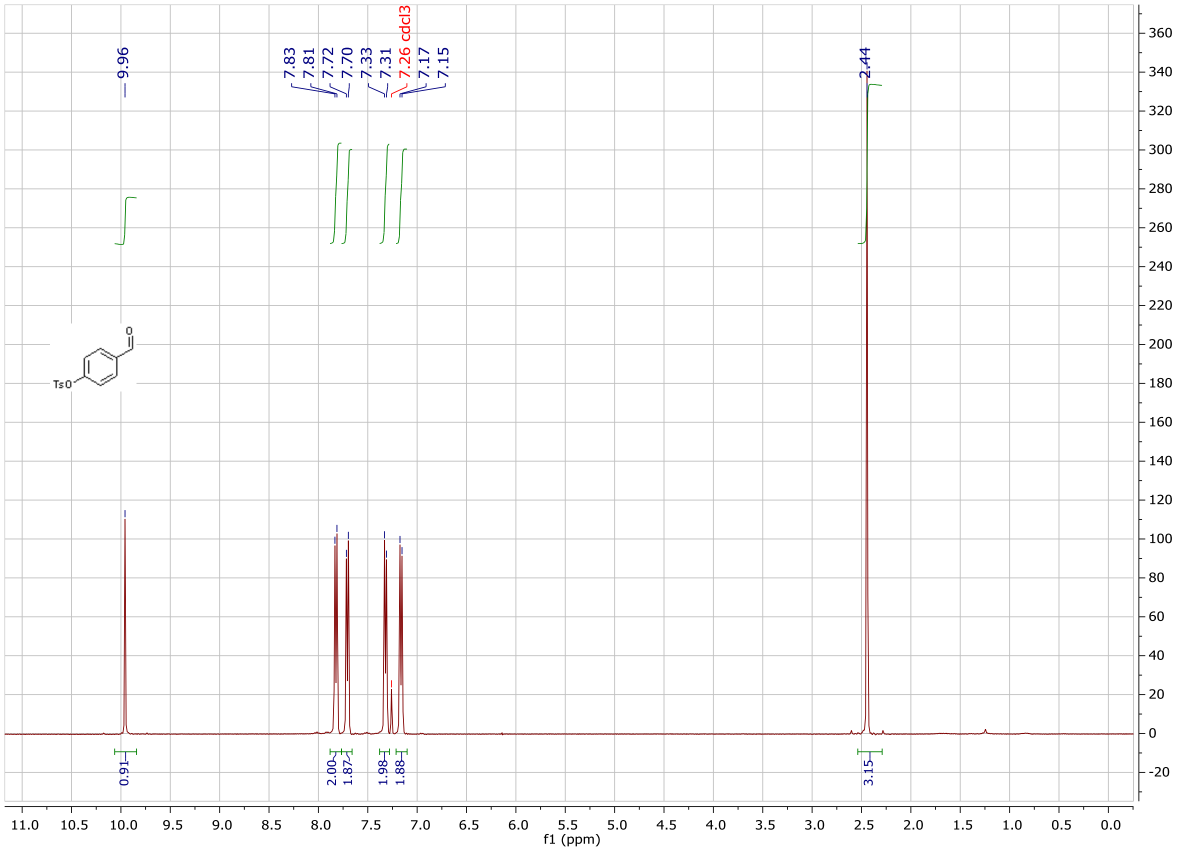

Figure S10. ^{1}H-NMR spectrum (400 MHz) of **15**

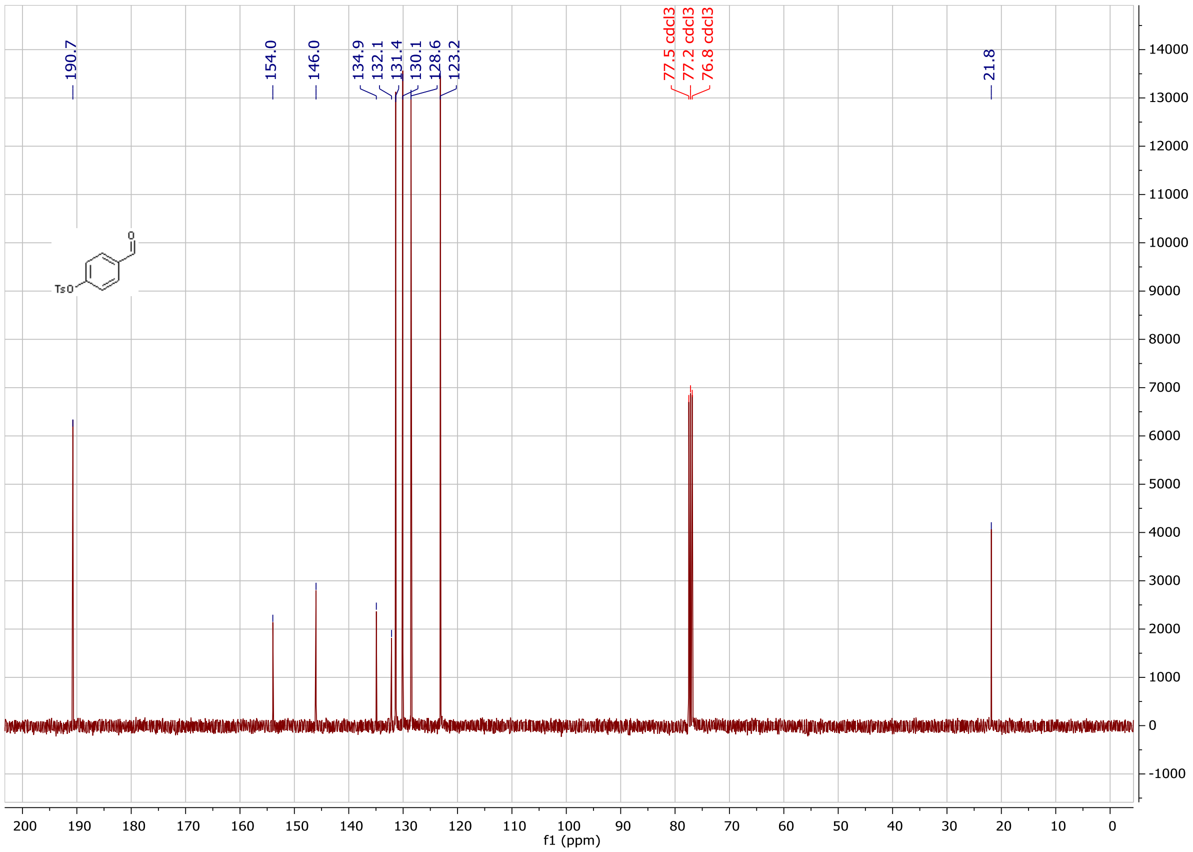

Figure S11. ^{13}C-NMR spectrum (101 MHz) of **15**

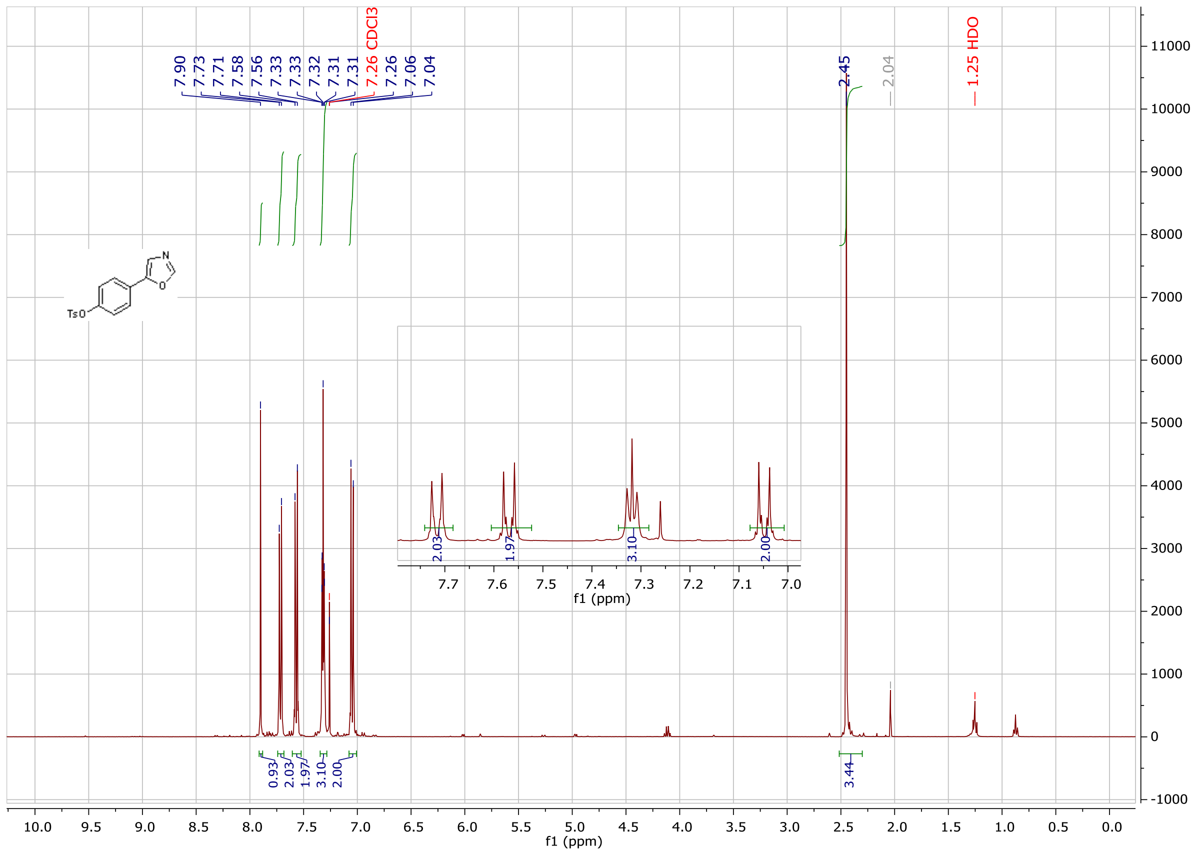

Figure S12. ^{1}H-NMR spectrum (400 MHz) of **16**

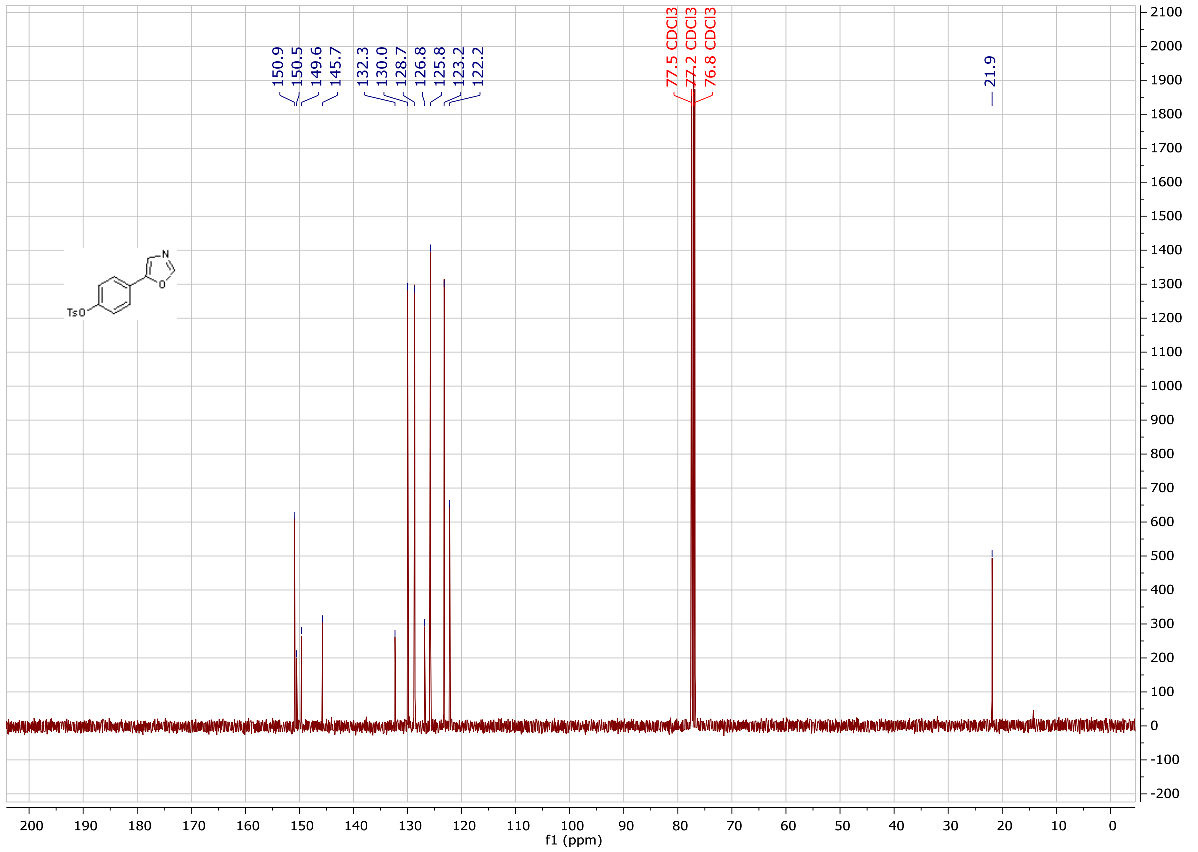

Figure S13. ^{13}C-NMR spectrum (101 MHz) of **16**

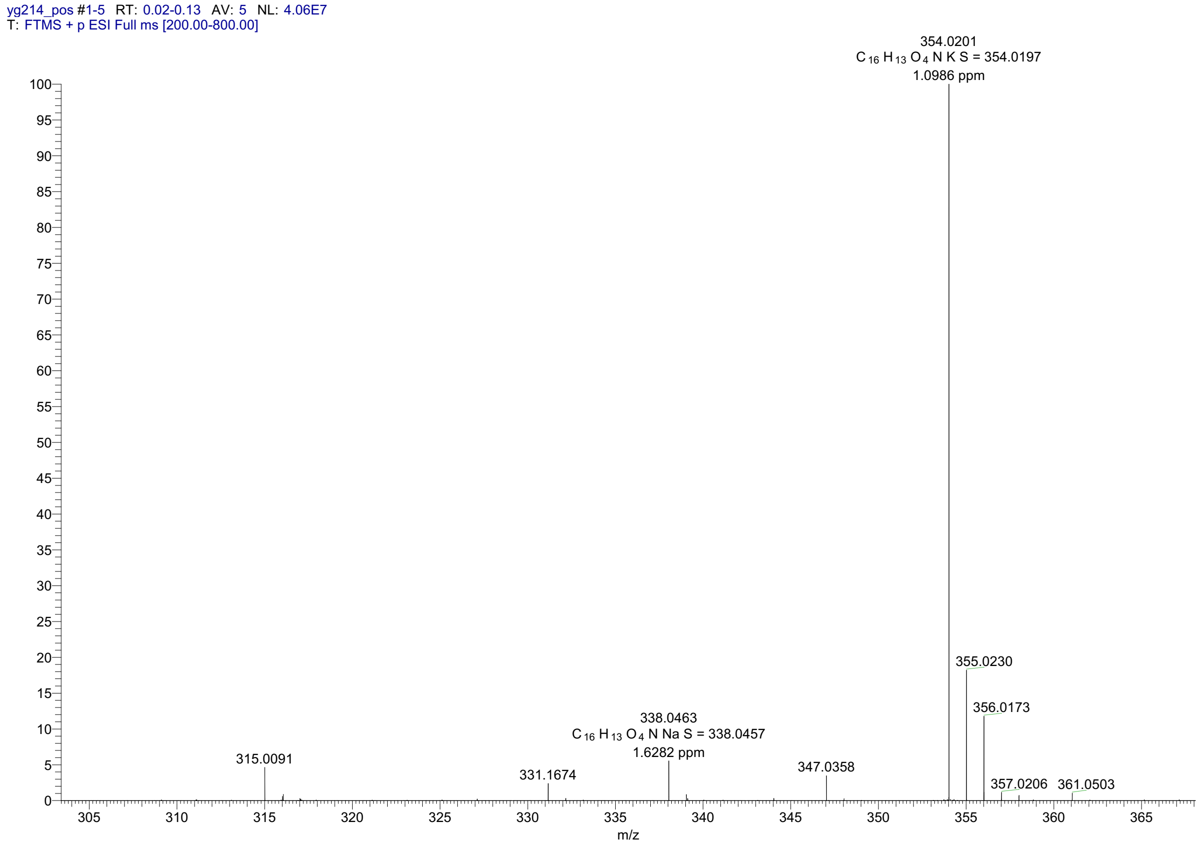

Figure S14.High resolution mass spectrum of **16.**

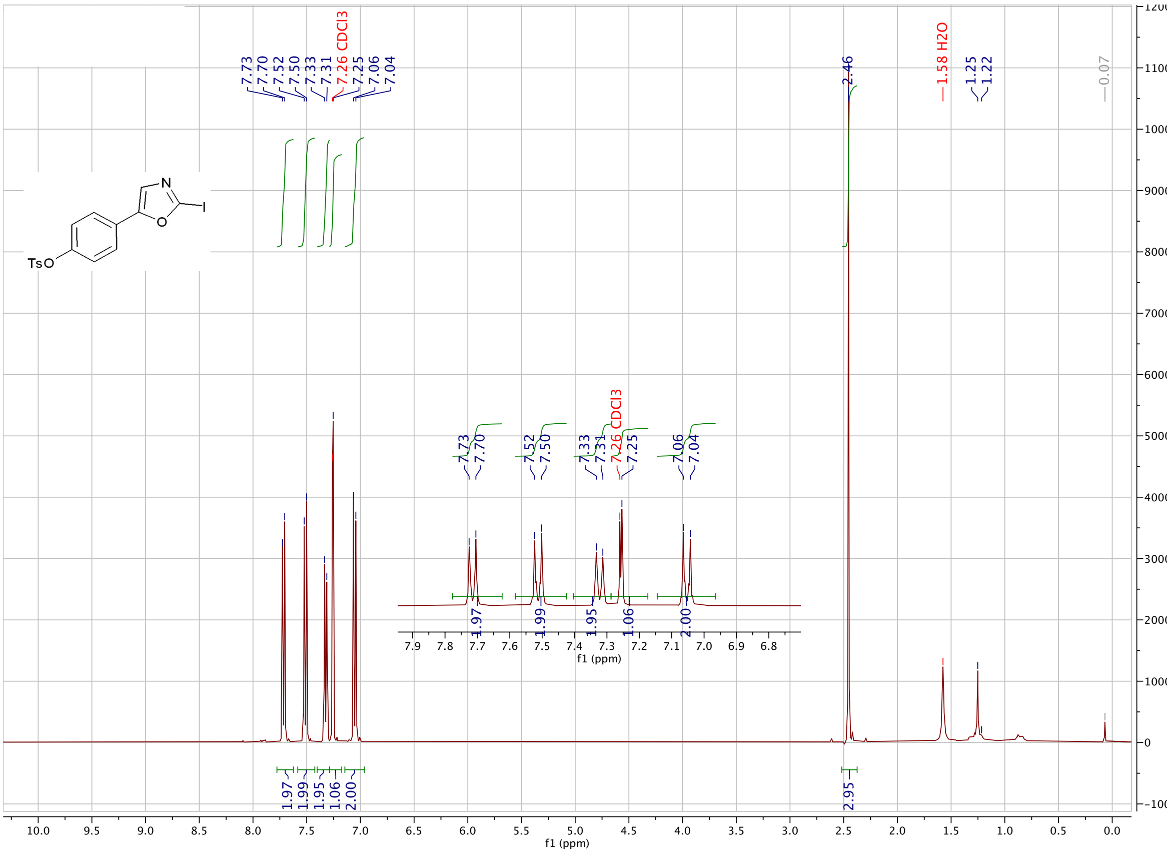

Figure S15. 1H-NMR spectrum (400 MHz) of **17**

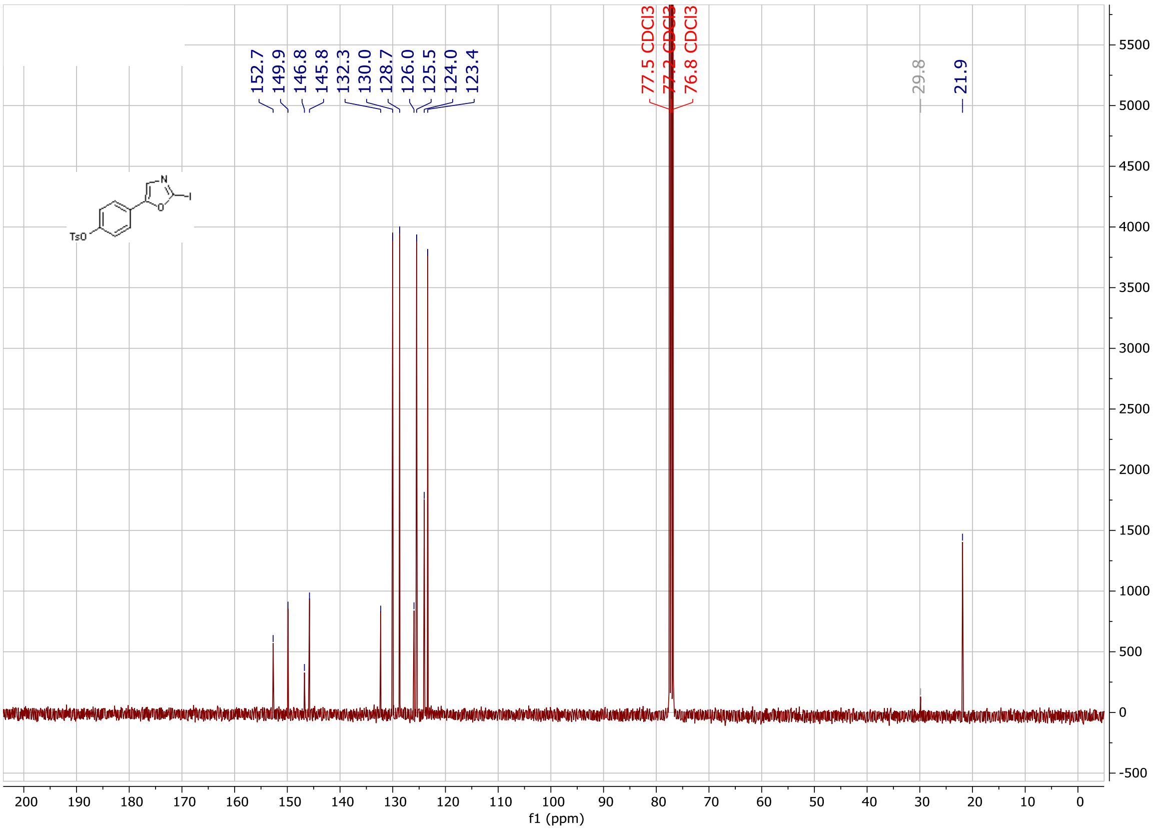

Figure S16. ^{13}C-NMR spectrum (101 MHz) of **17**

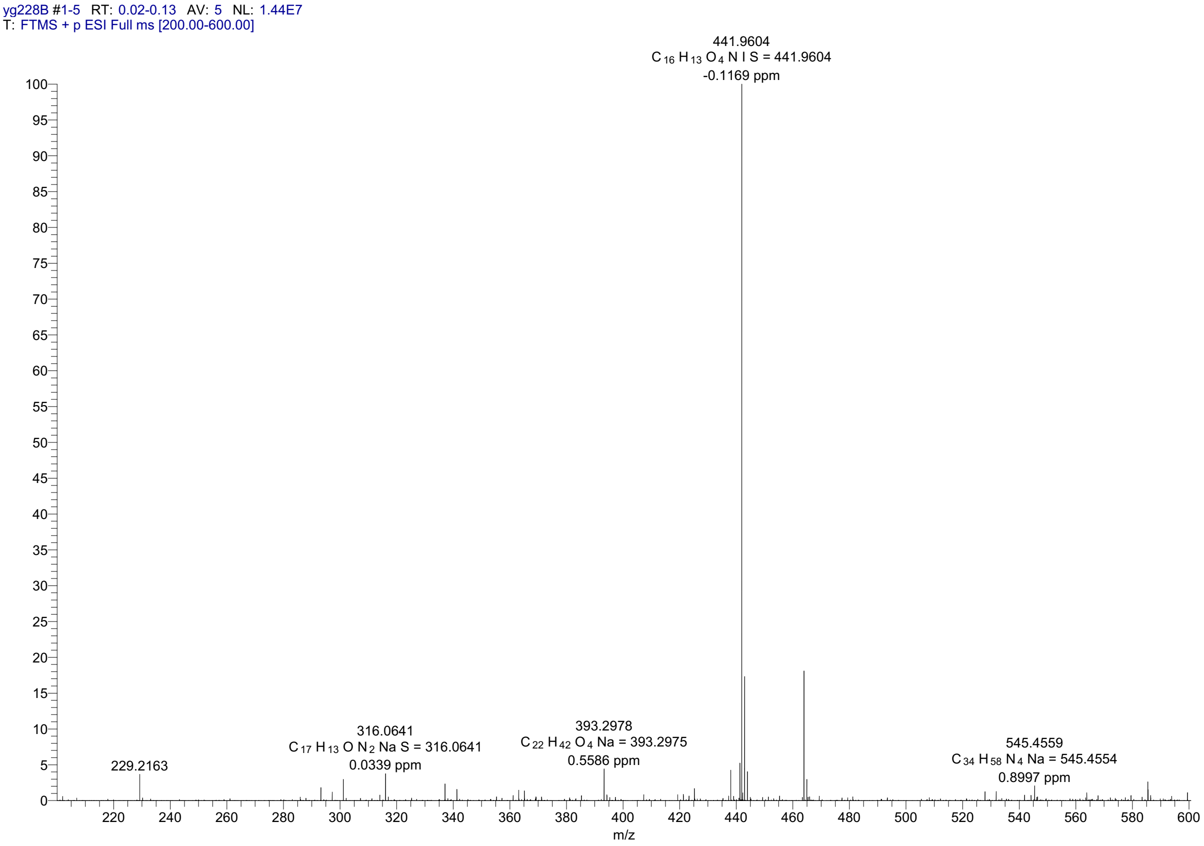

Figure S17.High resolution mass spectrum of **17.**

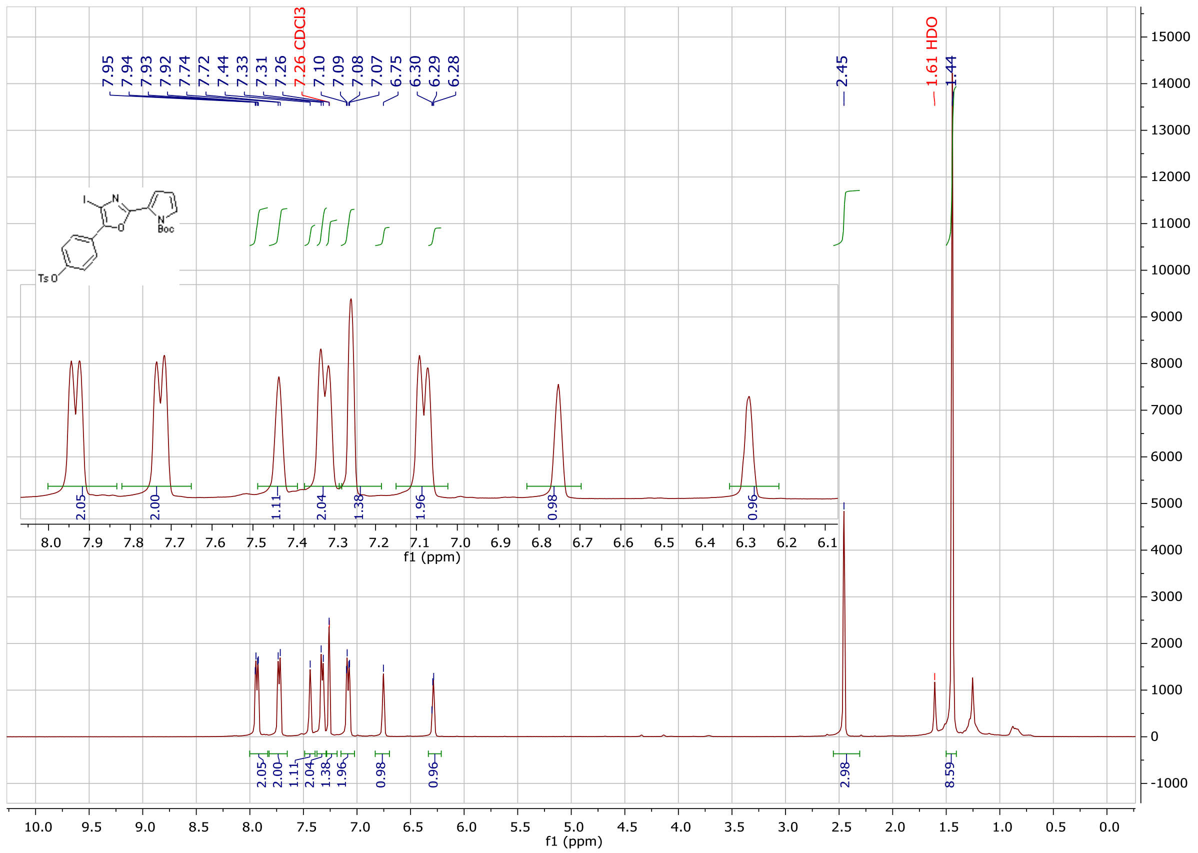

Figure S18. ^{1}H-NMR spectrum (400 MHz) of **18**

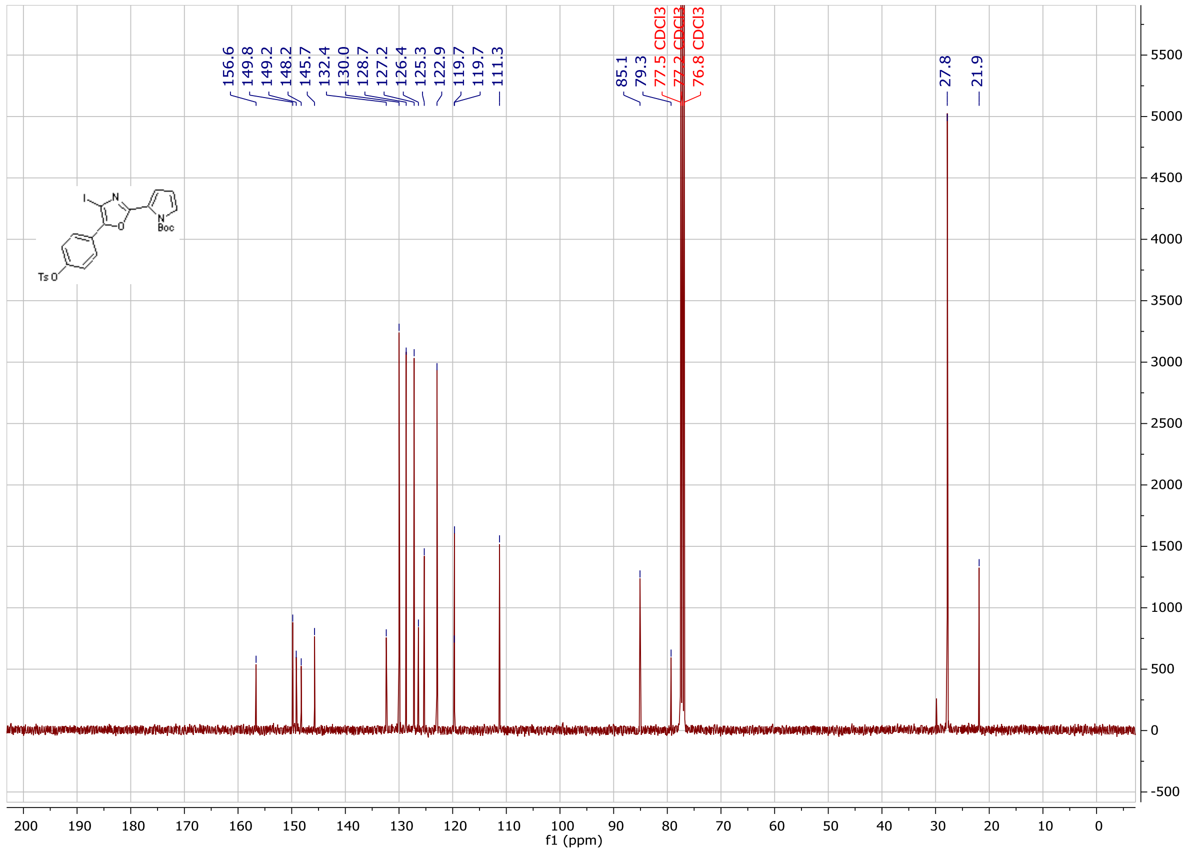

Figure S19. ^{13}C-NMR spectrum (101 MHz) of **18**

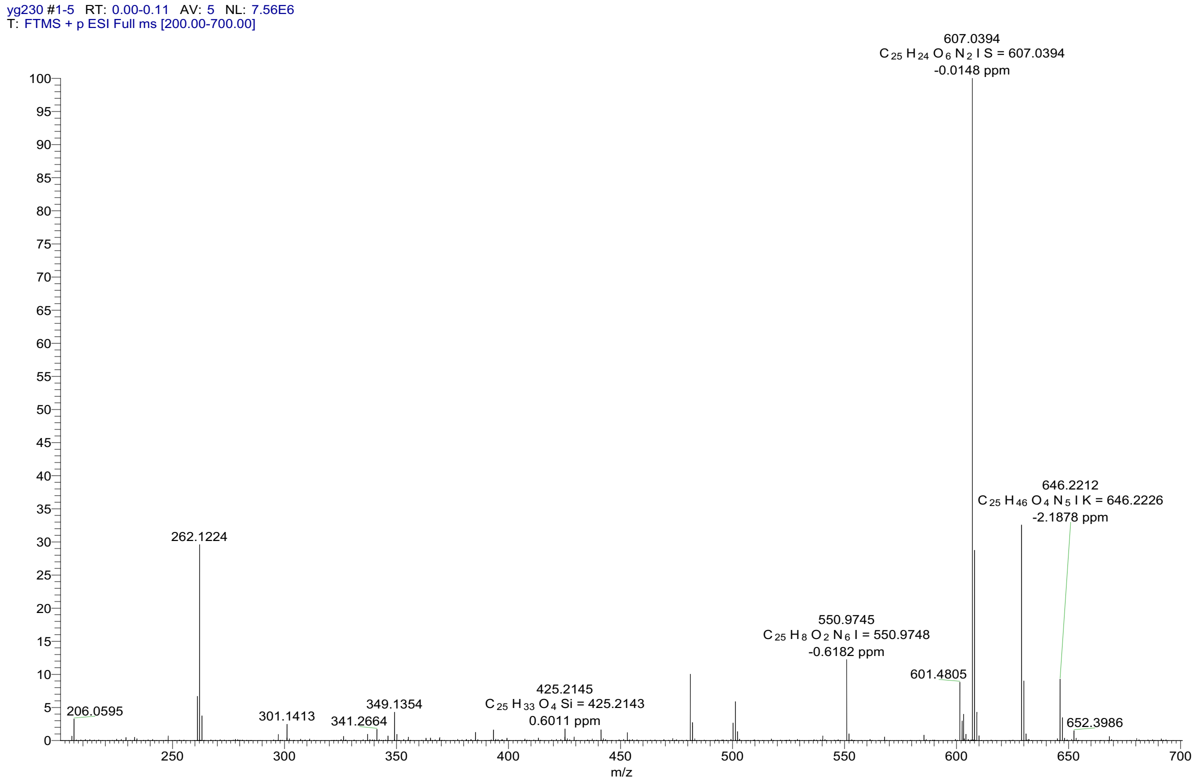

Figure S20.High resolution mass spectrum of **18.**

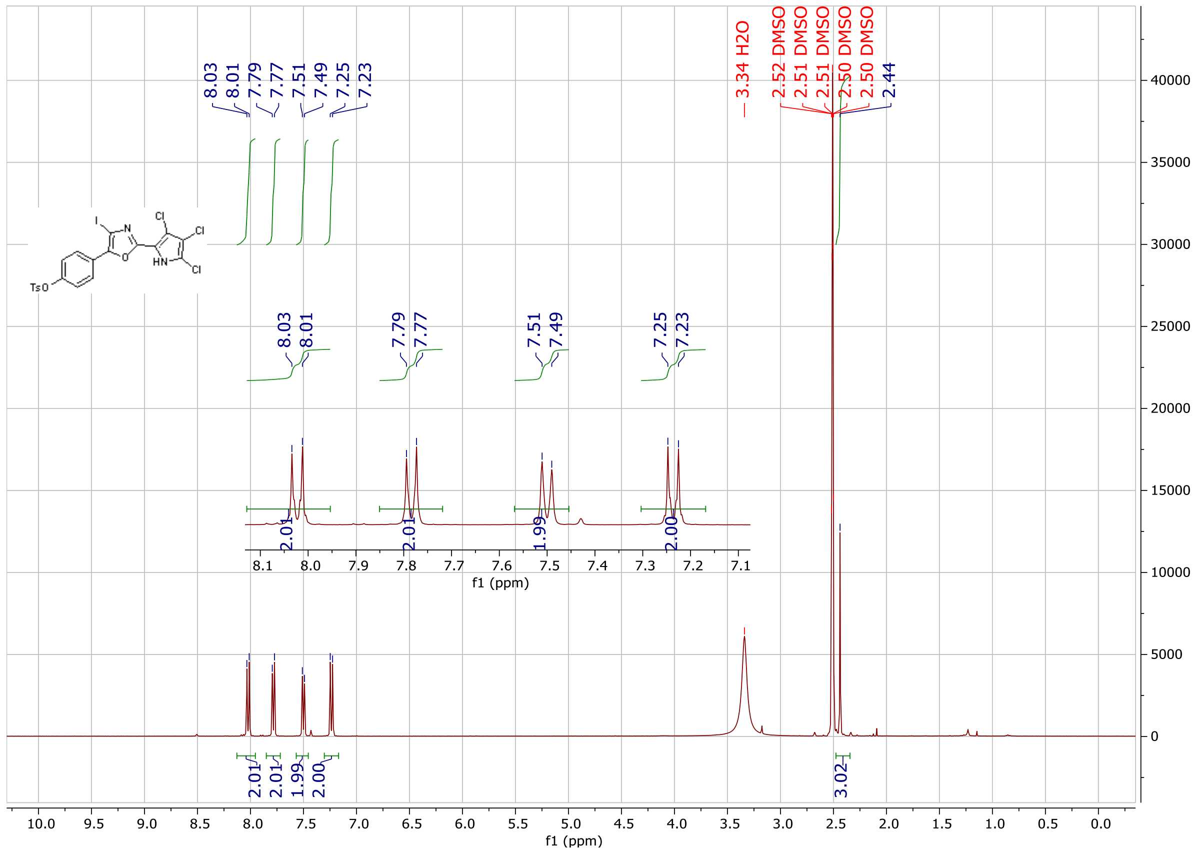

Figure S21. 1H-NMR spectrum (400 MHz) of **19**.

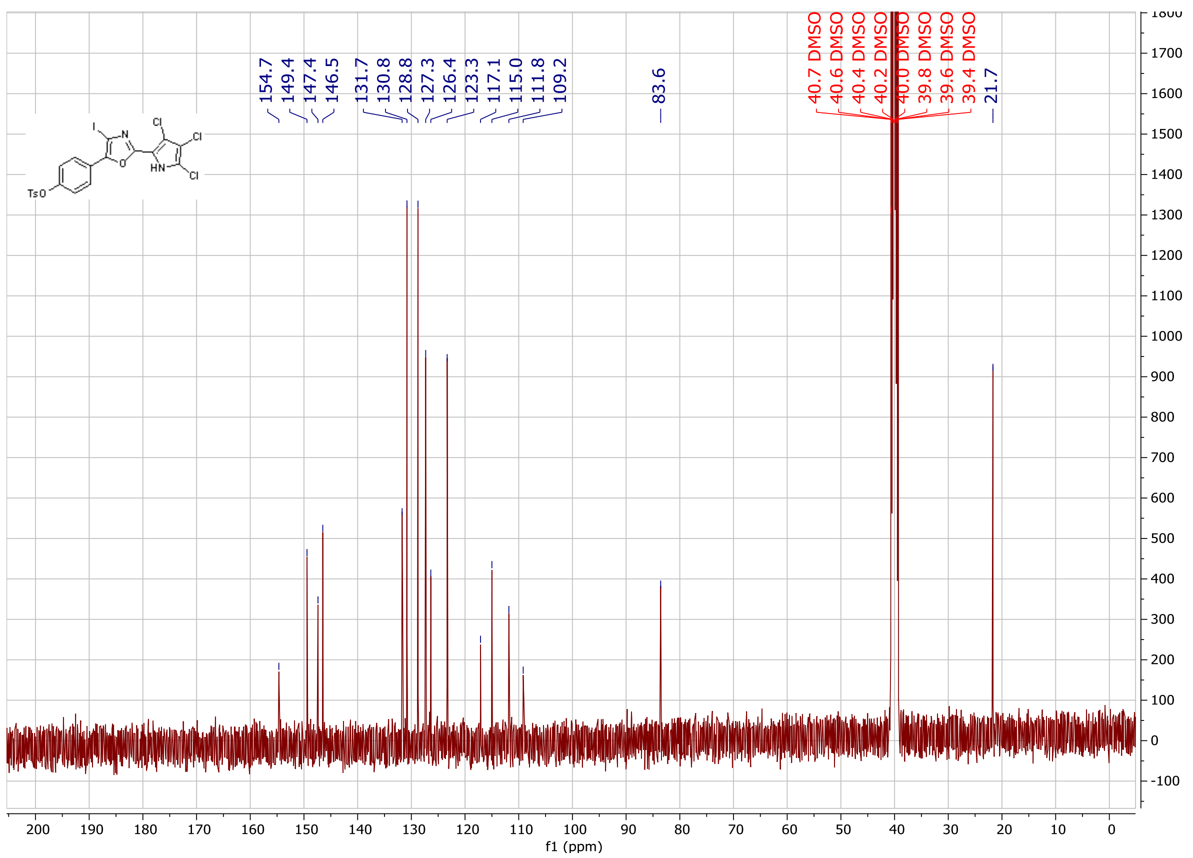

Figure S22. 13C-NMR spectrum (101 MHz) of **19**.

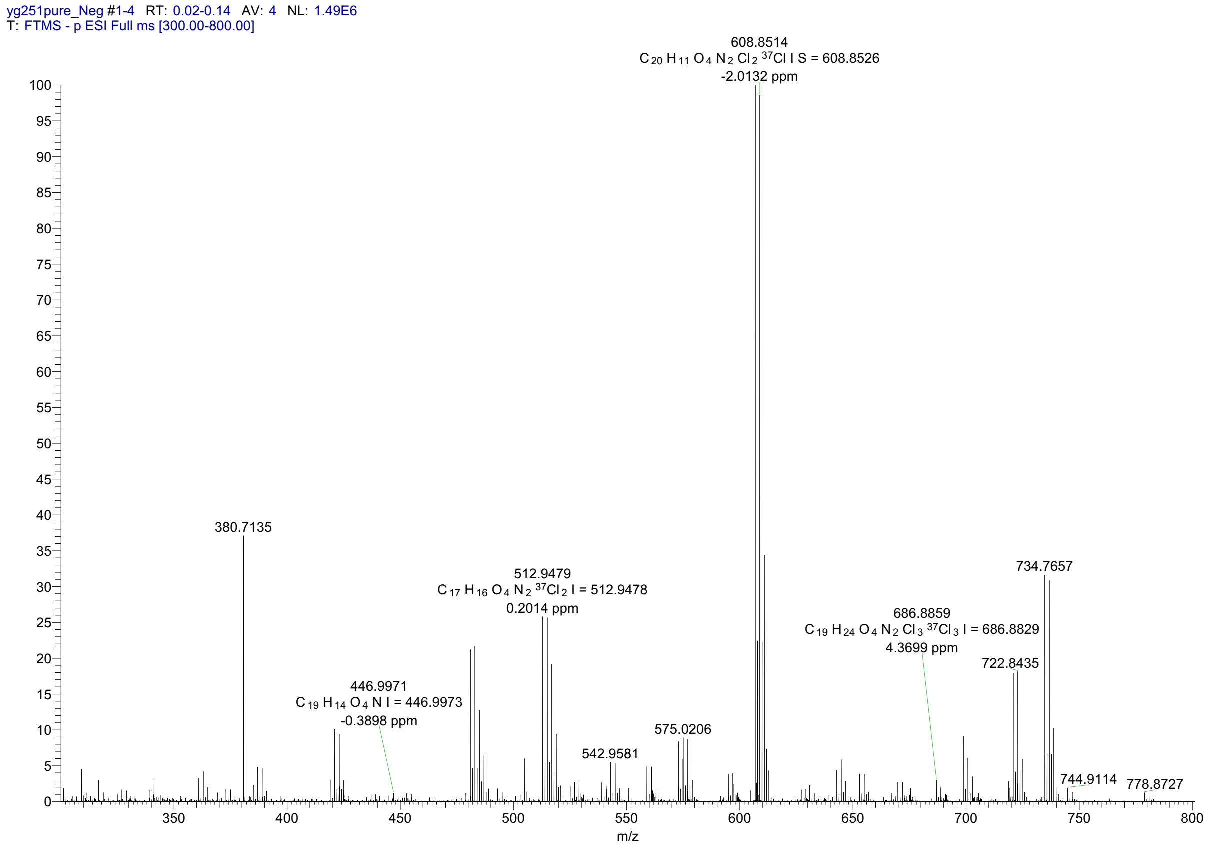

Figure S23.High resolution mass spectrum of **19.**

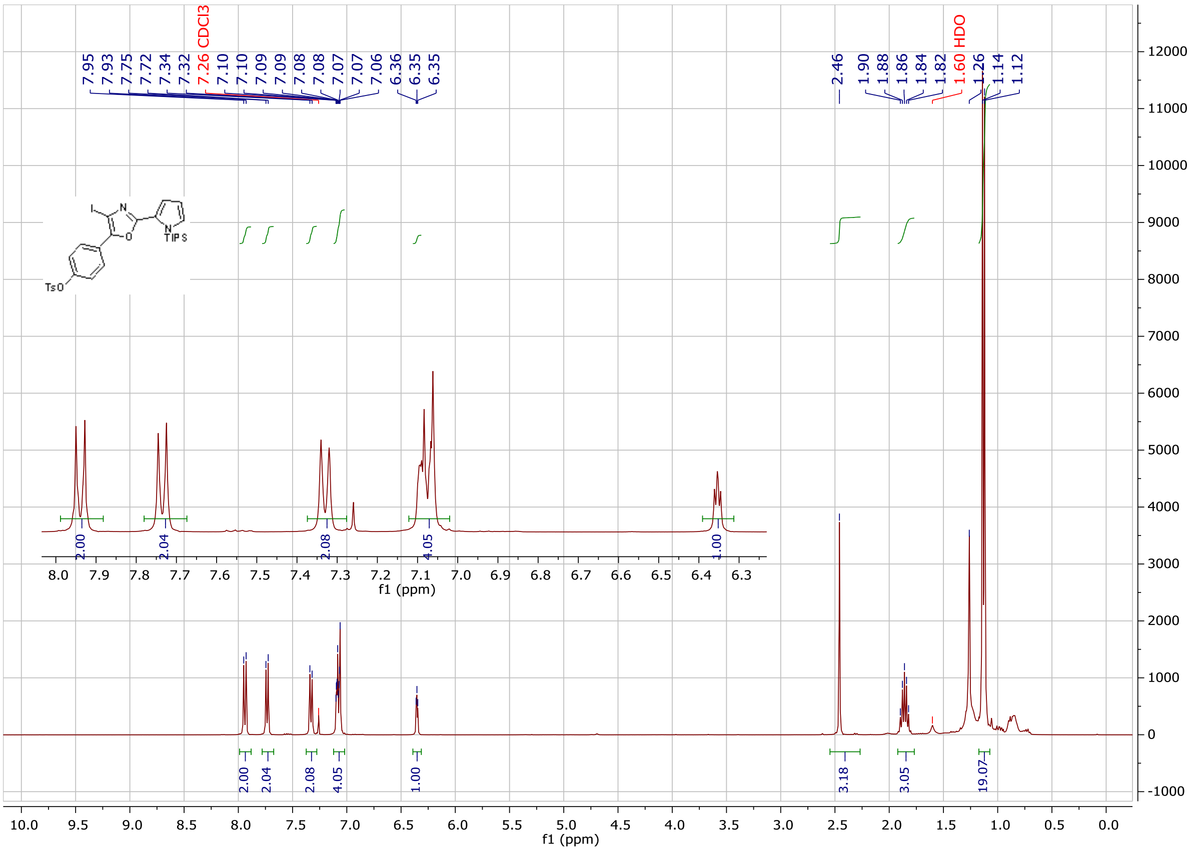

Figure S24. ^{1}H-NMR spectrum (400 MHz) of **20b**

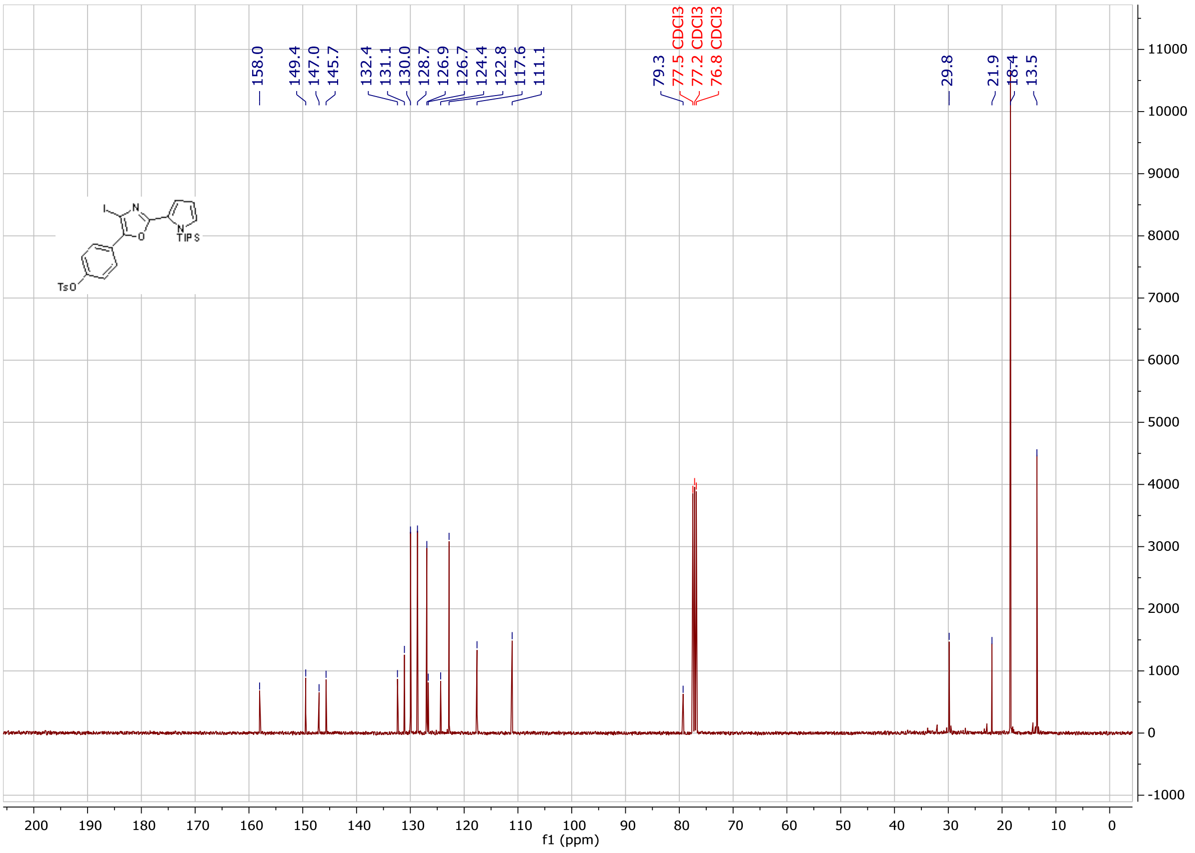

Figure S25. ^{13}C-NMR spectrum (101 MHz) of **20b**.

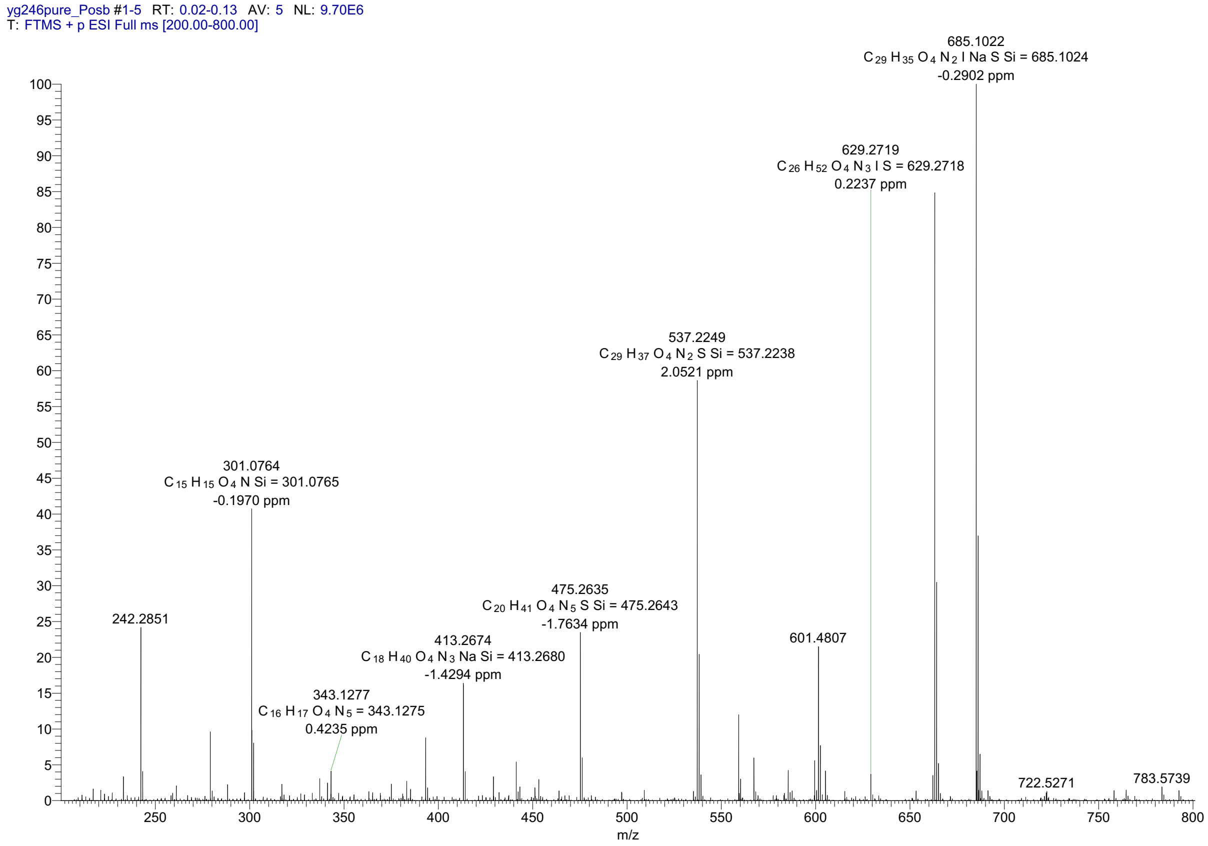

Figure S26. High resolution mass spectrum of **20b**

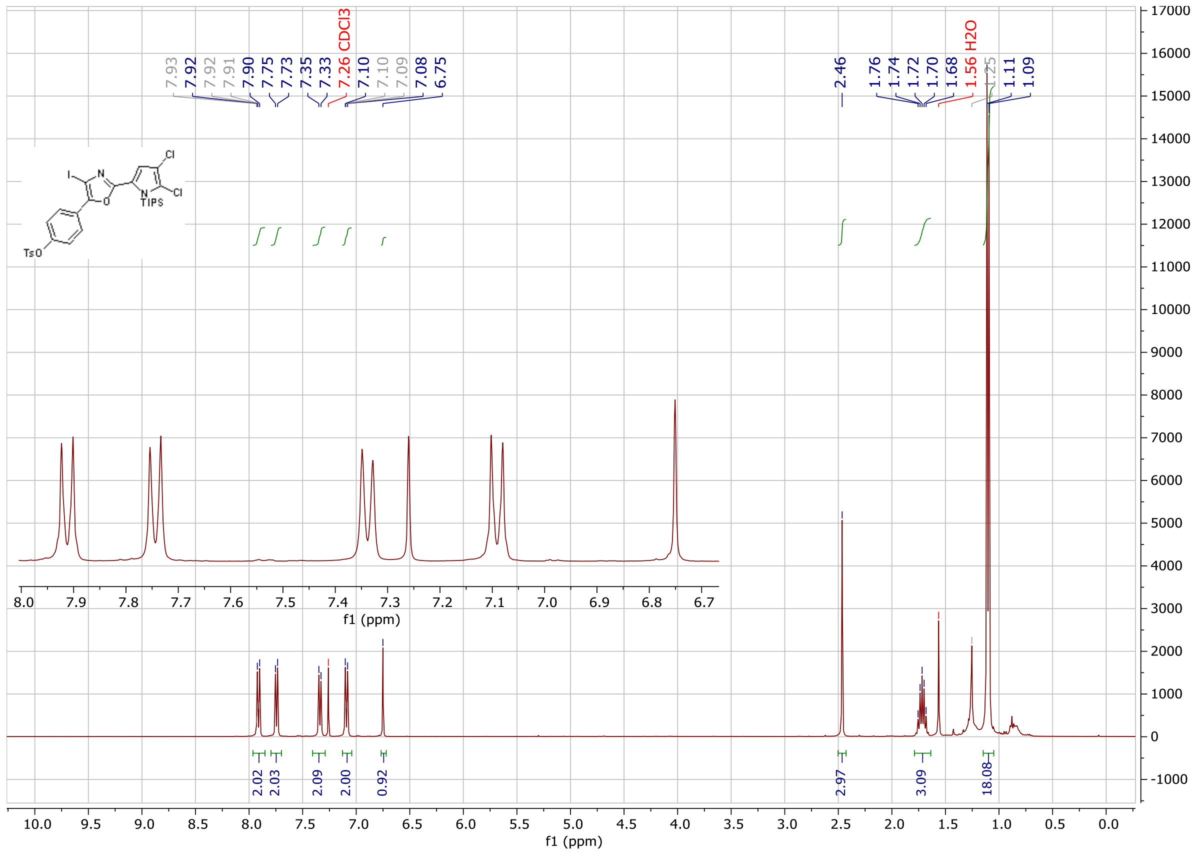

Figure S27. ^{1}H-NMR (400 MHz) spectrum of **21**.

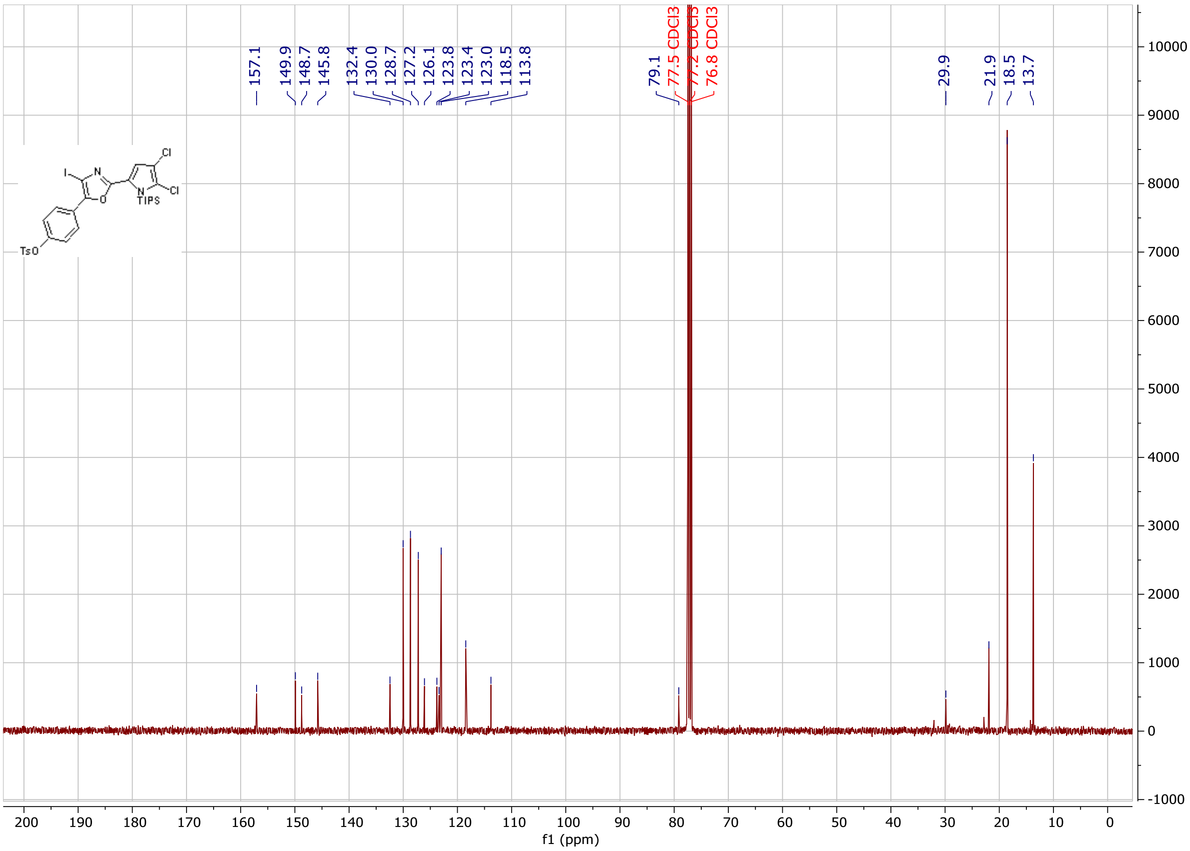

Figure S28. ^{13}C-NMR (101 MHz) spectrum of **21**.

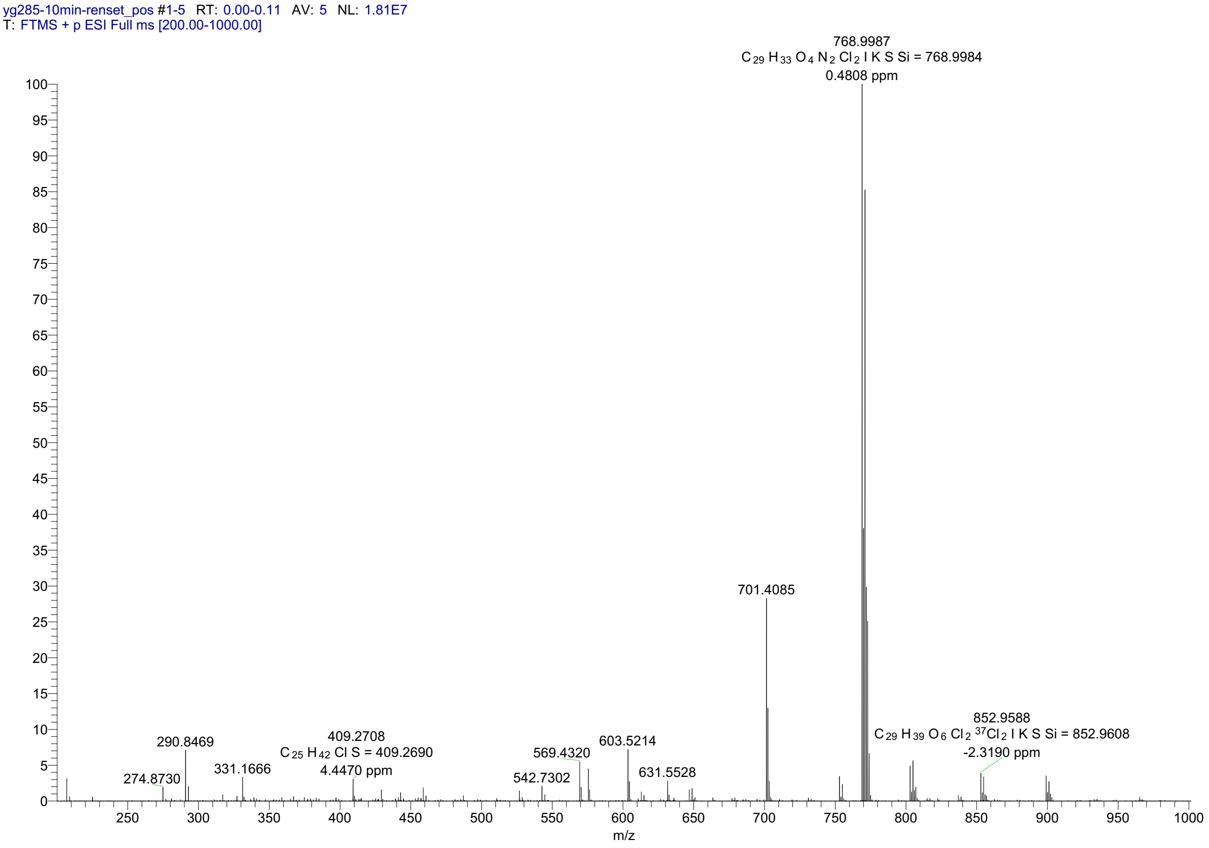

Figure S29. High resolution mass spectrum of **21**.

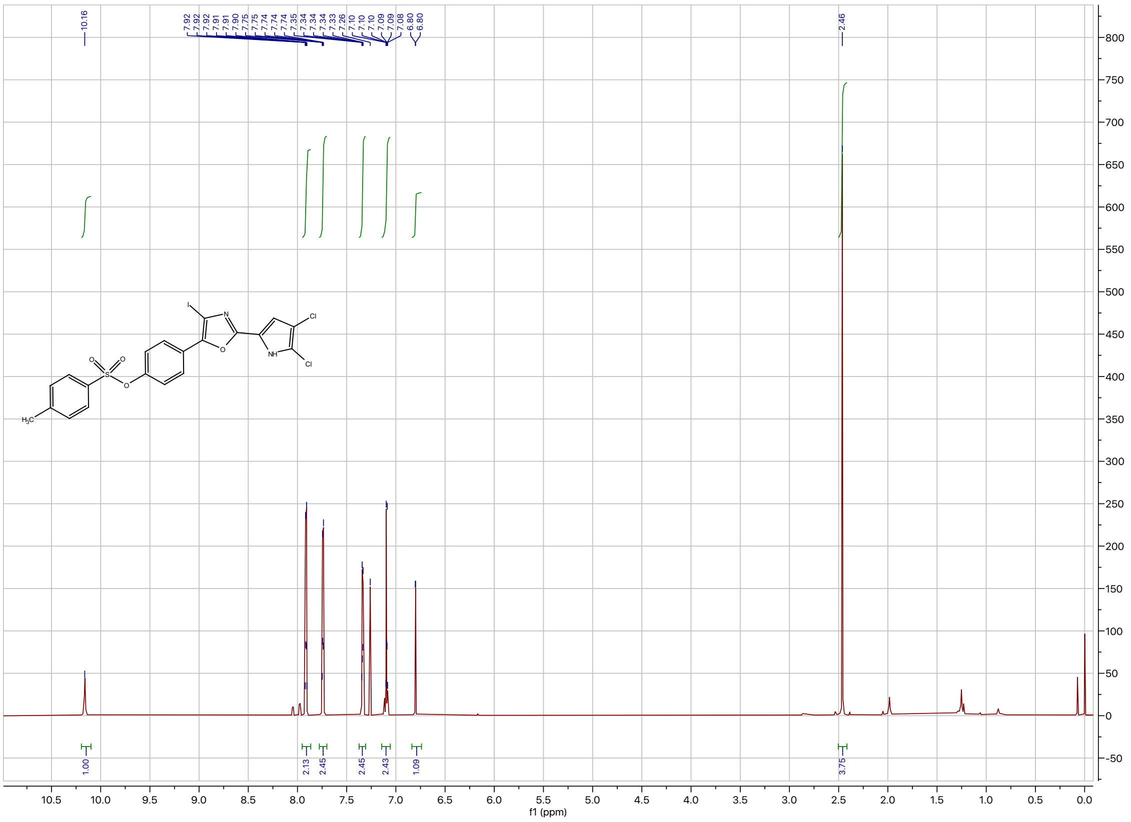

Figure S30. ^{1}H-NMR (850 MHz) spectrum of **22**.

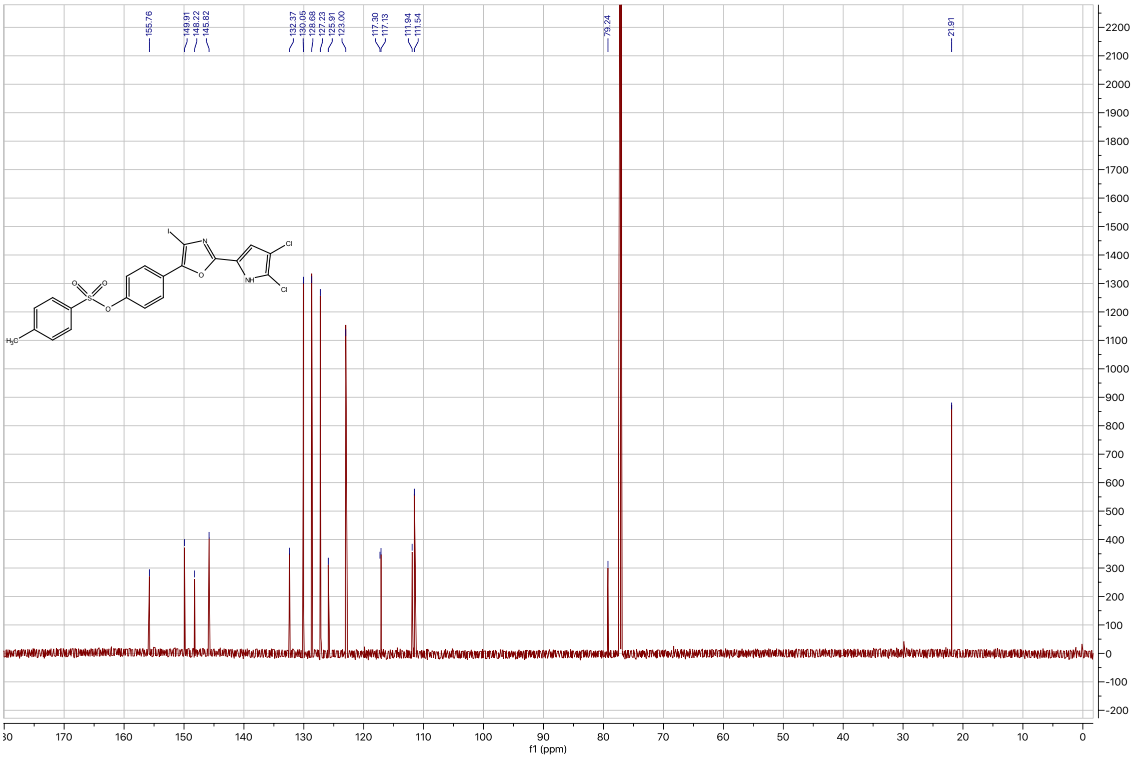

Figure S31. ^{13}C-NMR (126 MHz) spectrum of **22**.

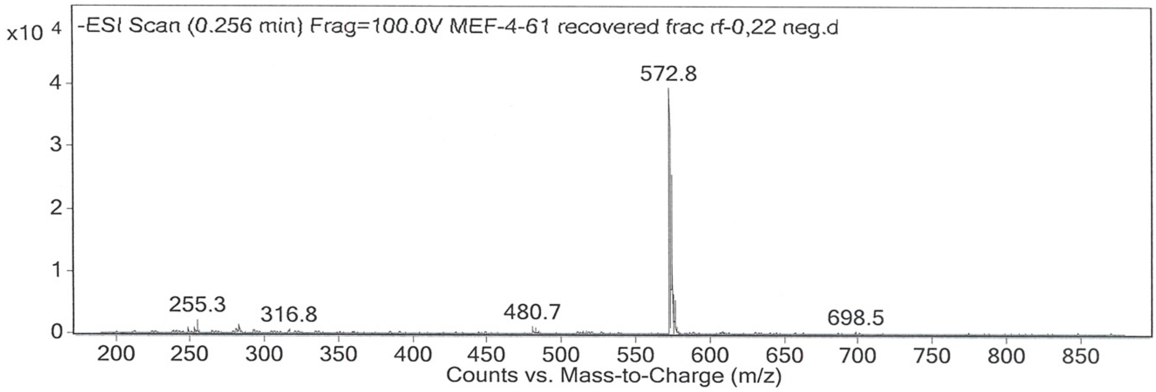

Figure S32. Low resolution MS of **22**.

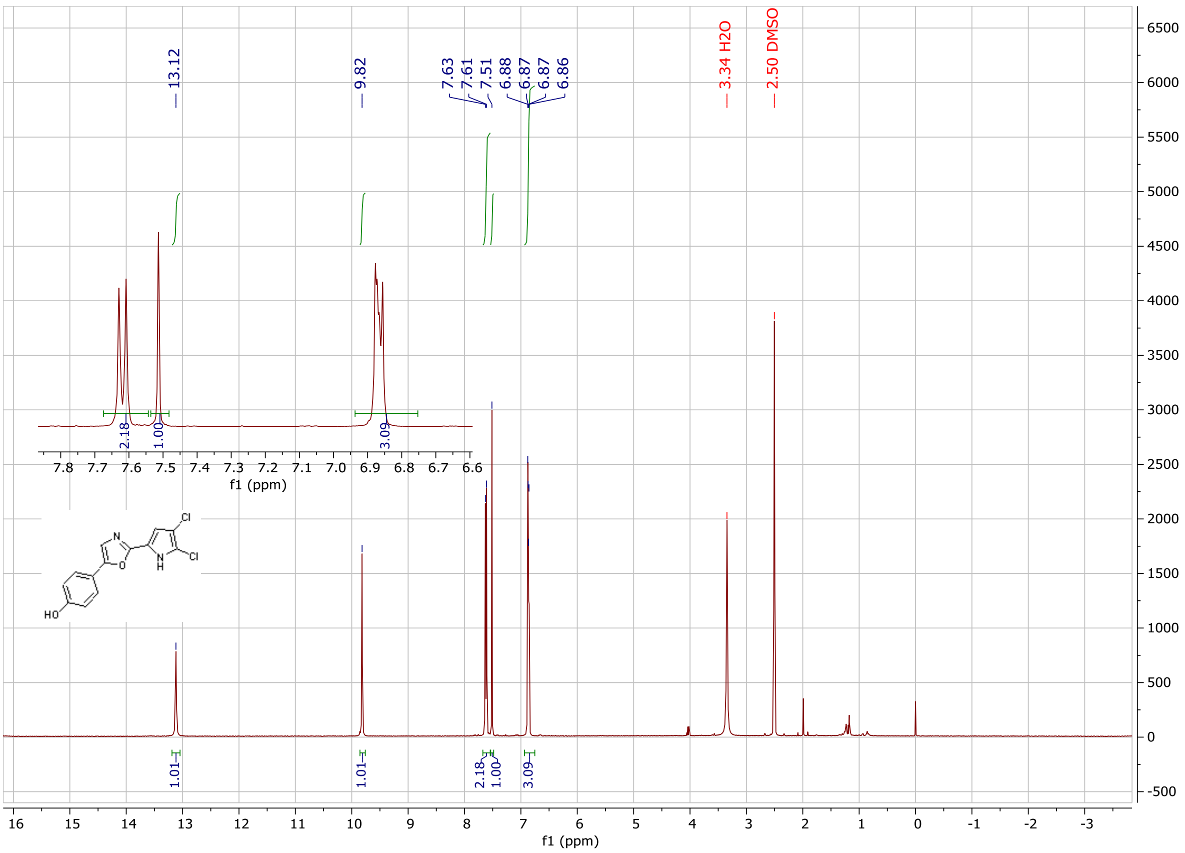

Figure S33. ^{1}H-NMR (400 MHz) spectrum of **23**.

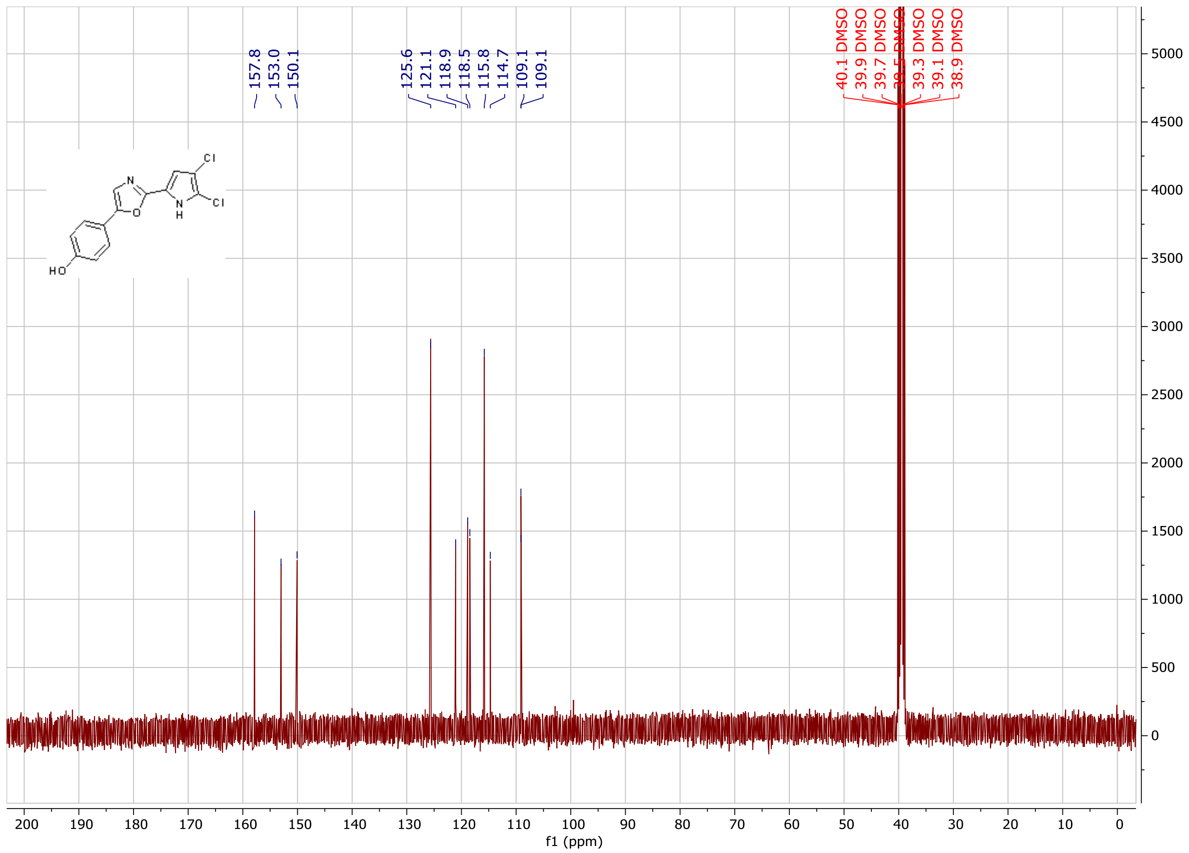

Figure S34. ^{13}C-NMR (101 MHz) spectrum of **23**.

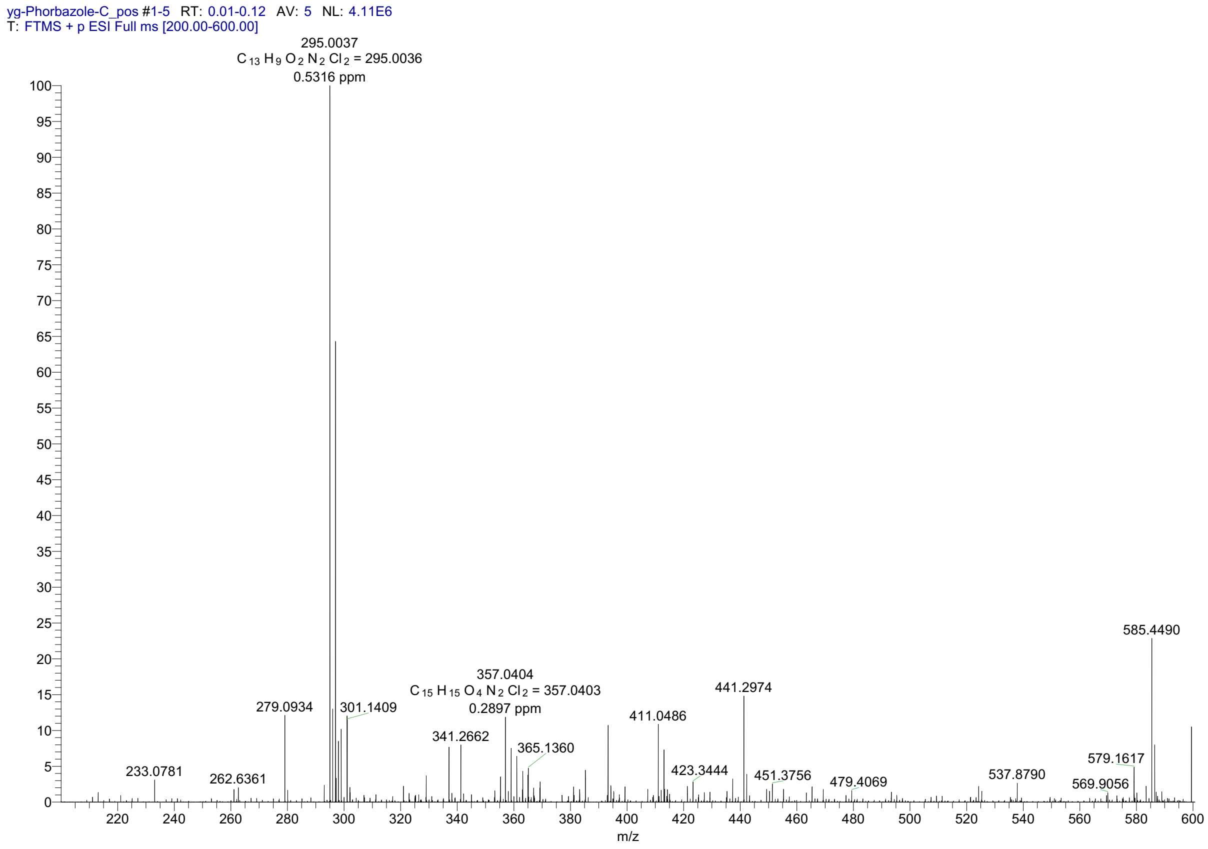

Figure S35.High resolution mass spectrum of **23**.

Synthesis of 4-(2-(4,5-Dichloro-1H-pyrrol-2-yl)oxazol-5-yl)phenyl 4-methylbenzenesulfonate (**24**)

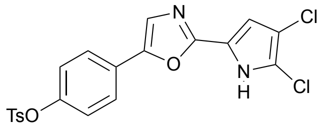

Dichlorinated pyrrole **21** (20 mg, 27 µmol) was dissolved in ethanol (0.5 mL) and concentrated HCl (50 µL) was added. A precipitate formed immediately, but dissolved again within one minute. After 5 minutes, zinc (18 mg, 0.27 mmol) was added and the reaction mixture was heated at reflux for 2 h. The reaction mixture was cooled to rt and then adsorbed onto a Biotage snaplet precolumn, evaporated and purified by flash chromatography using a Biotage SNAP Ultra column using 0-100 % ethyl acetate in heptane to give the title compound.

Colourless solid; yield 5 mg (41 %); mp.: 188-192°C (dec); ^{1}H-NMR (400 MHz, $SO(CD_3)_2$) δ = 11.95 (s, 1H), 7.79 (m, 4H), 7.63 (s, 1H), 7.50 (d, *J* = 7.9, 2H), 7.14 (d, *J* = 8.6, 2H), 6.93 (s, 1H), 2.49 (s, 3H); ^{13}C-NMR (101 MHz, $CO(CD_3)_2$) δ = 149.3, 149.1, 146.0, 132.3, 130.1, 128.5, 126.9, 125.3, 124.1, 123.1, 110.4, 109.9, 20.7 (three more peaks visible in HMBC); HRMS (ESI) *m/z* calcd. for $C_{20}H_{13}O_4N_2Cl_2S$ $[M-H]^-$: 446.9973; found: 466.9969.

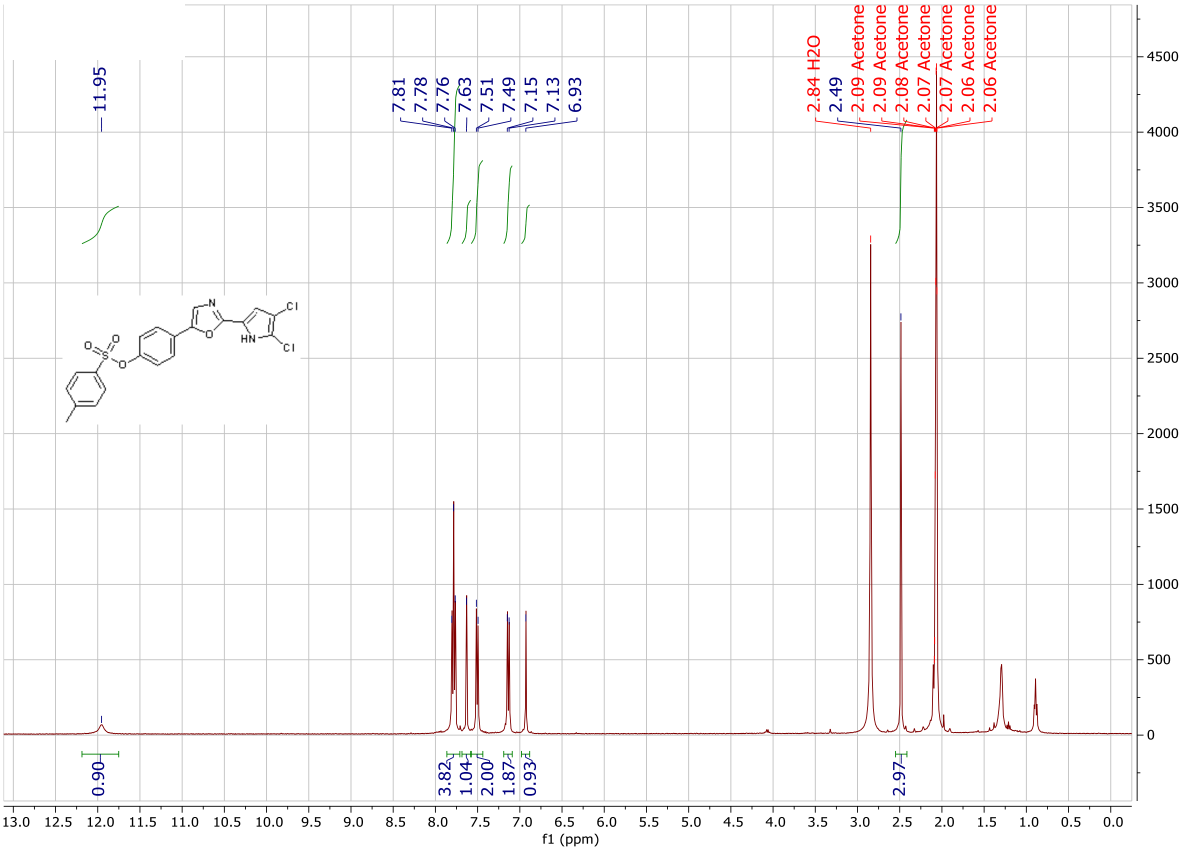

Figure S36. ^{1}H-NMR (400 MHz) spectrum of **24**.

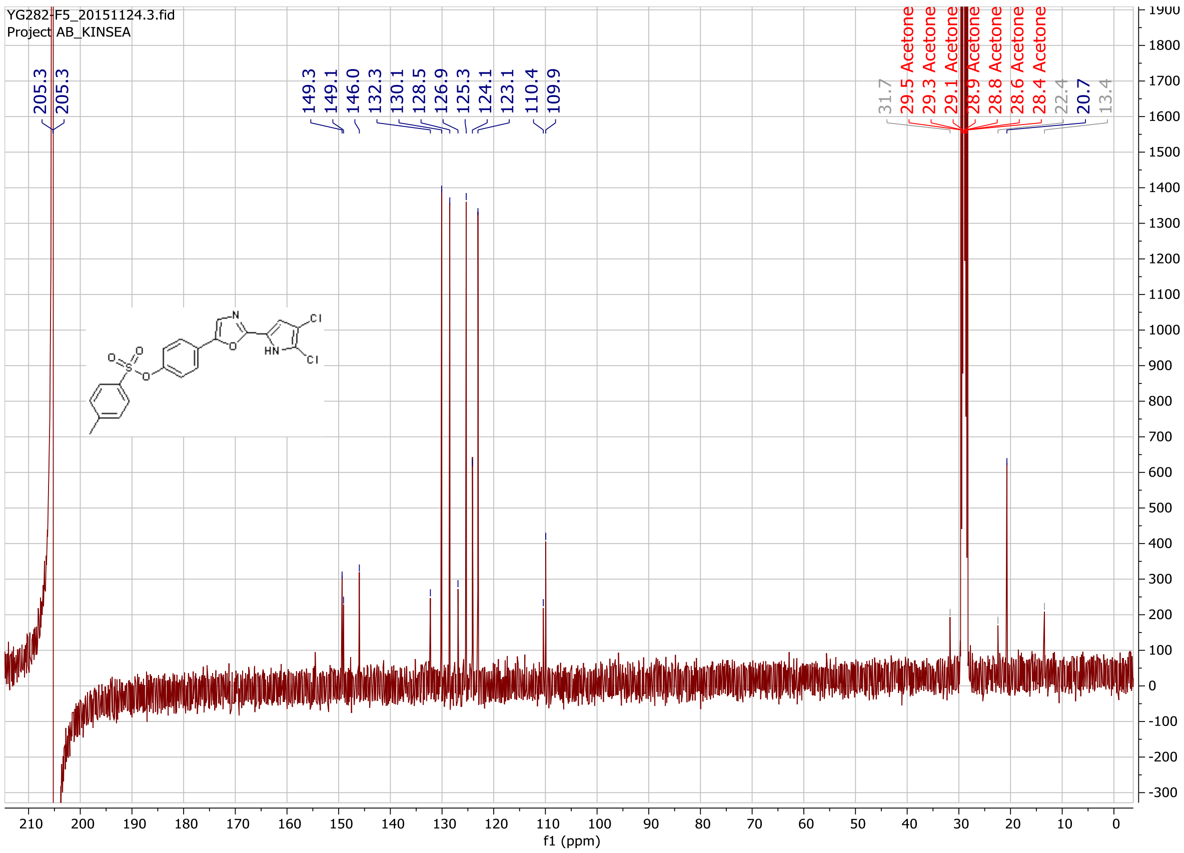

Figure S37. ^{13}C-NMR (101 MHz) spectrum of **24**.

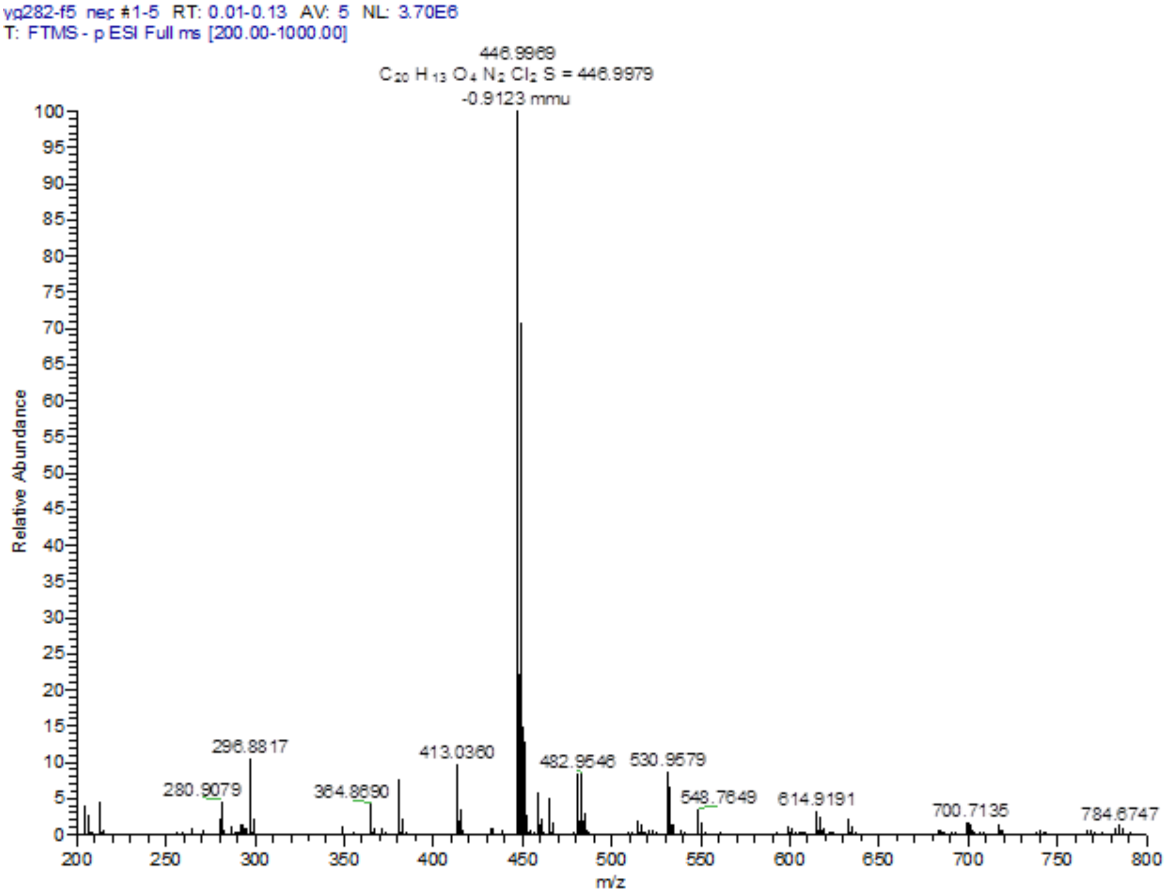

Figure S35.High resolution mass spectrum of **24**.

Structural assignment for **21** and **24**

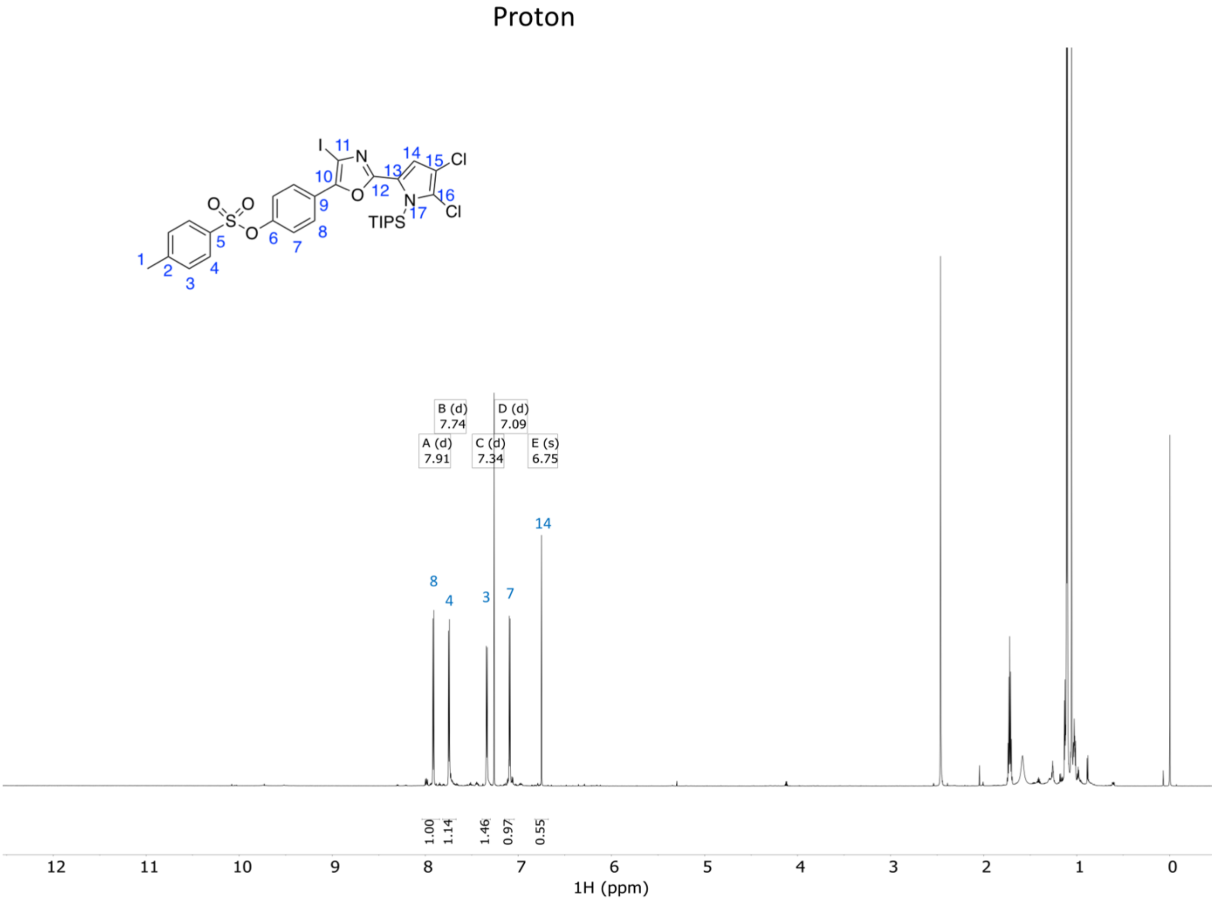

Figure S36. ^{1}H-NMR (850 MHz) spectrum of **21**.

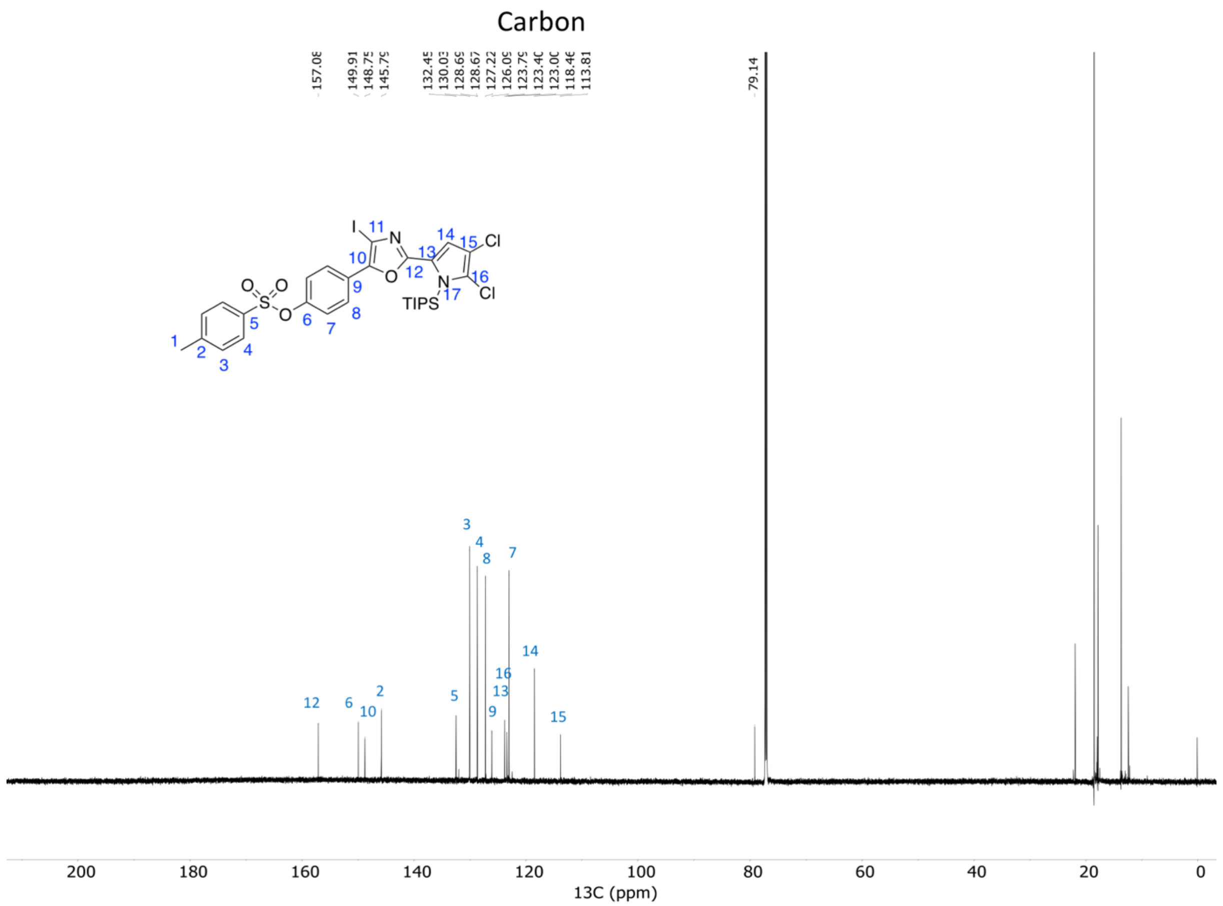

Figure S37. ^{13}C-NMR (214 MHz) spectrum of **21.**

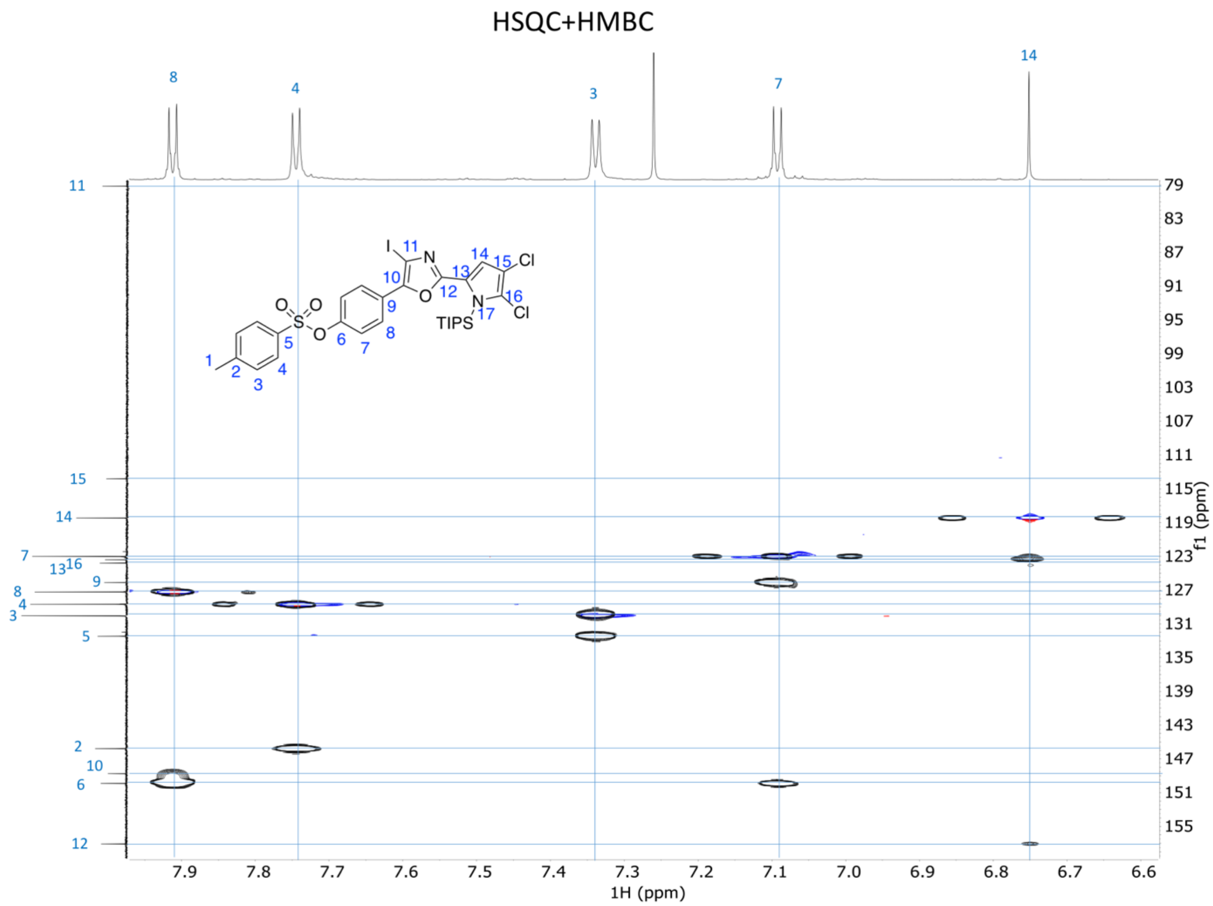

Figure S38. Superimposed HSQC and HMBC of **21**.

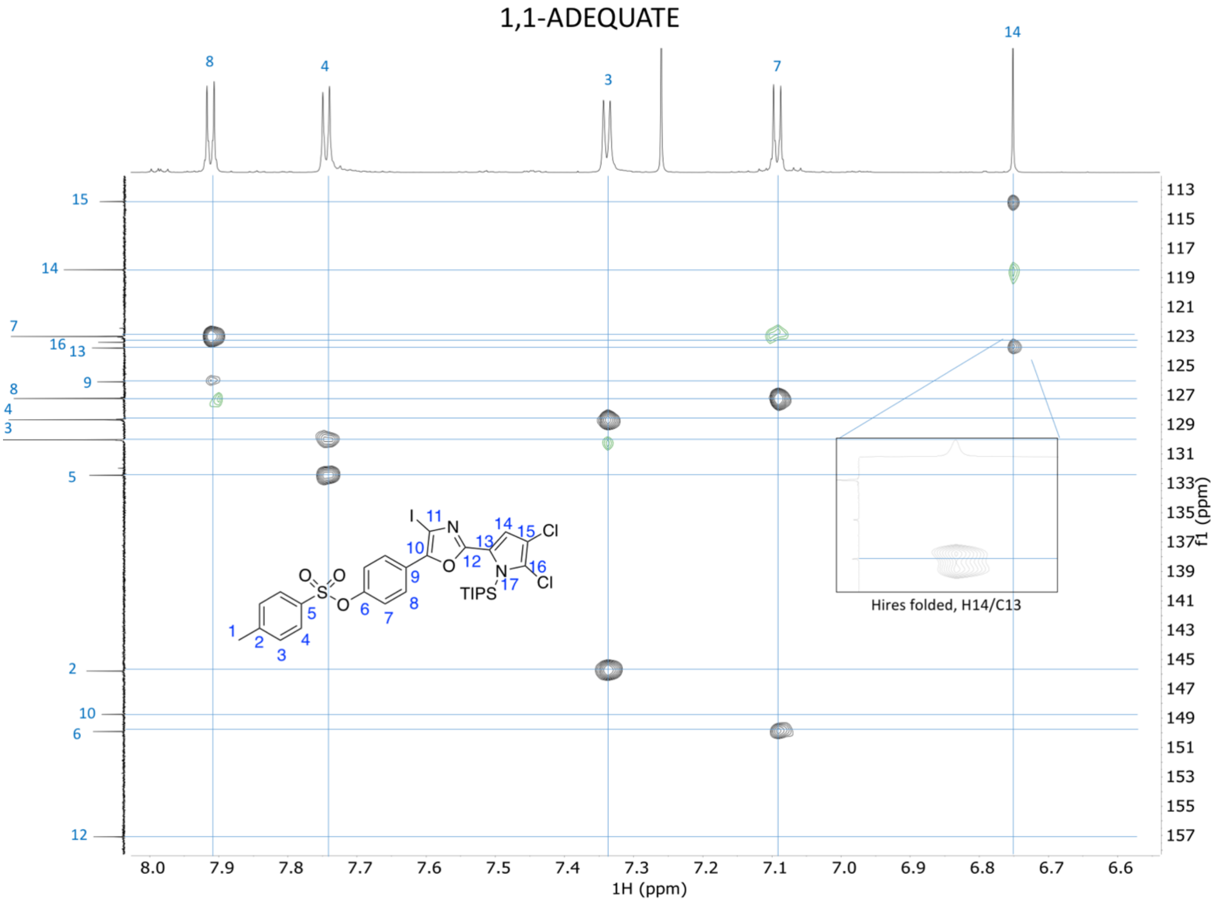

Figure S39. 1,1-ADEQUATE of **21**.

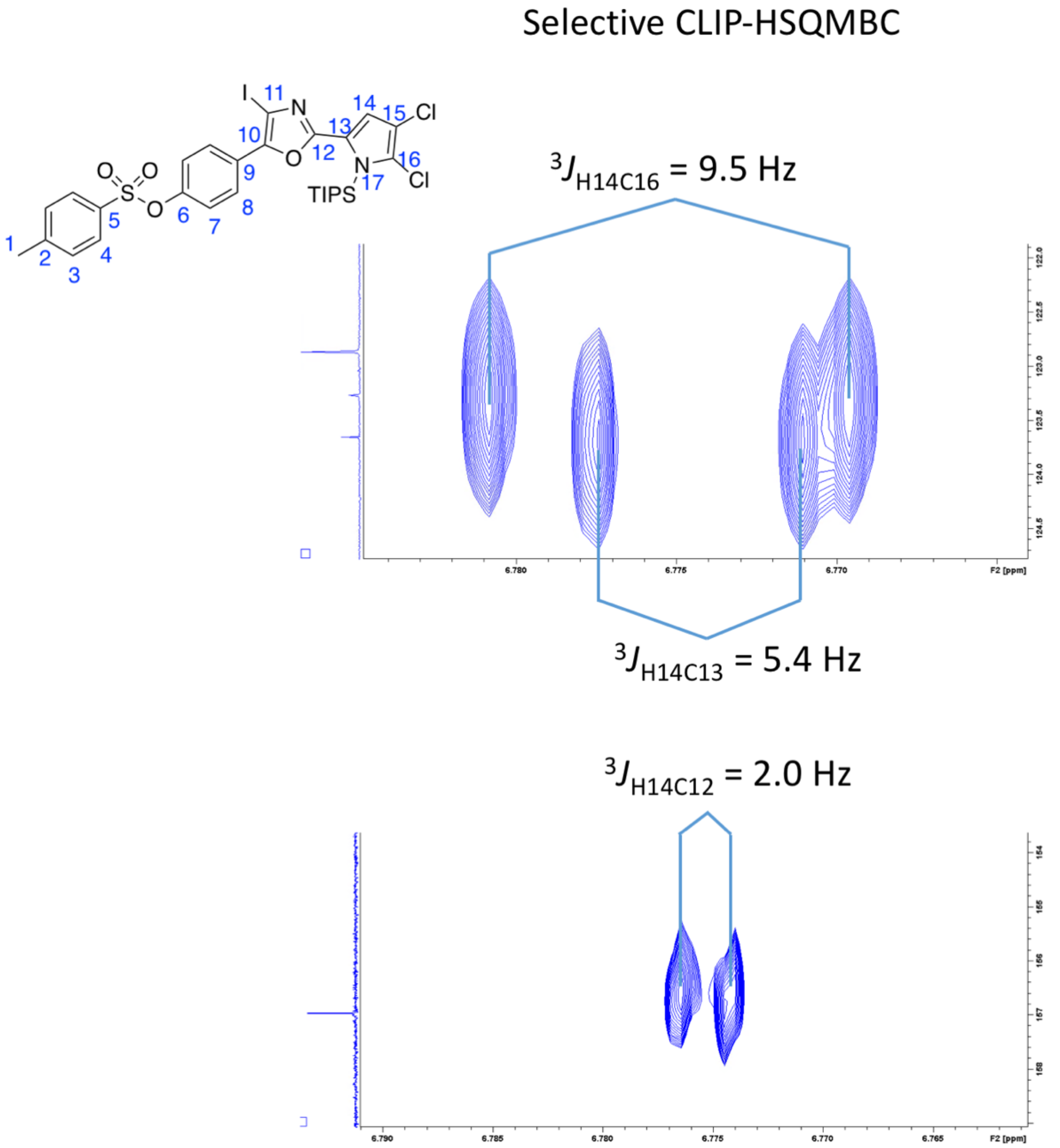

Figure S40. Selective CLIP-HSQMBC spectra of **21**.

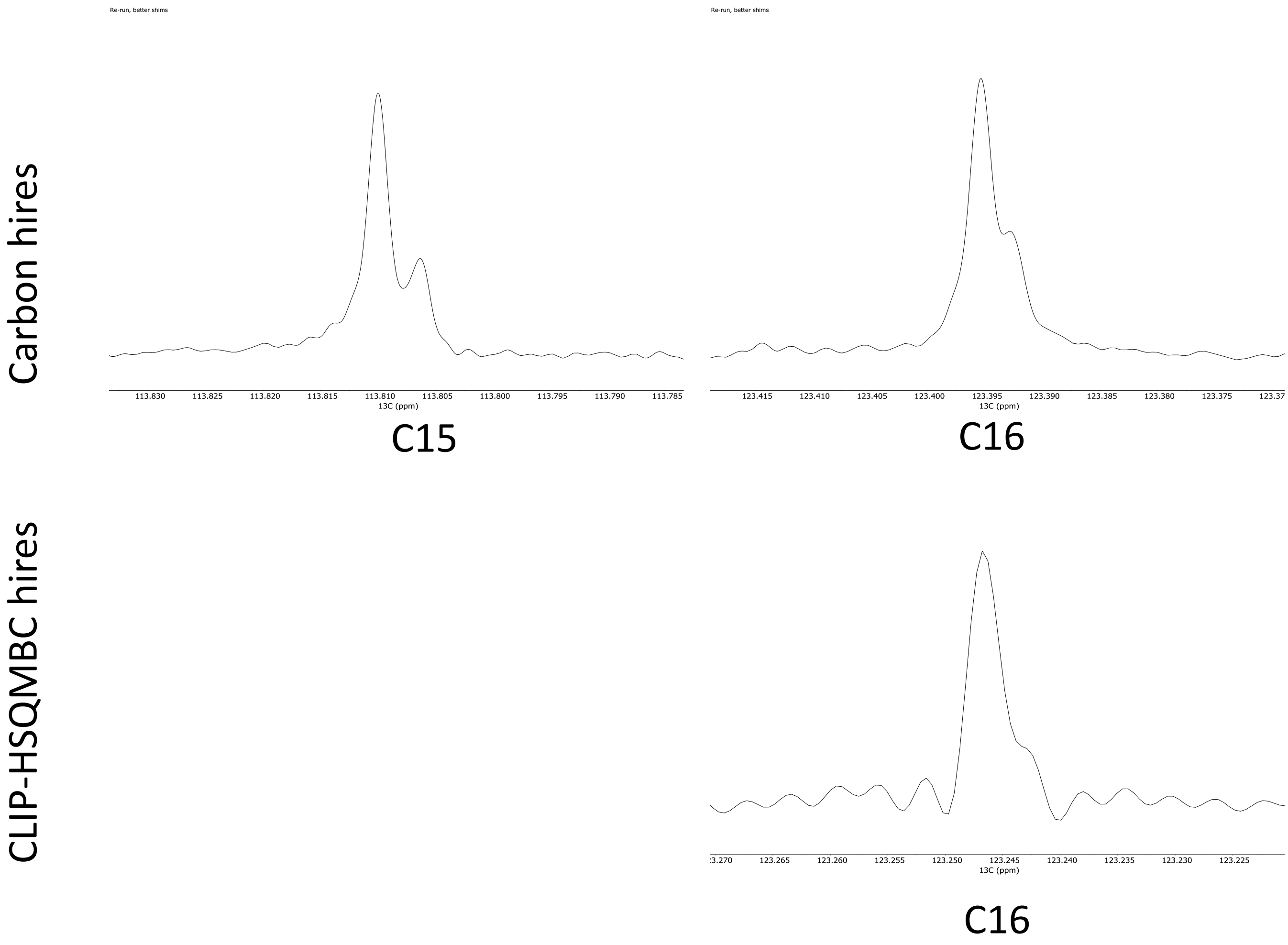

Figure S41. Hires ^{13}C (top panel) and hires selective CLIP-HSQMBC f1 projection (bottom panel) of **21**.

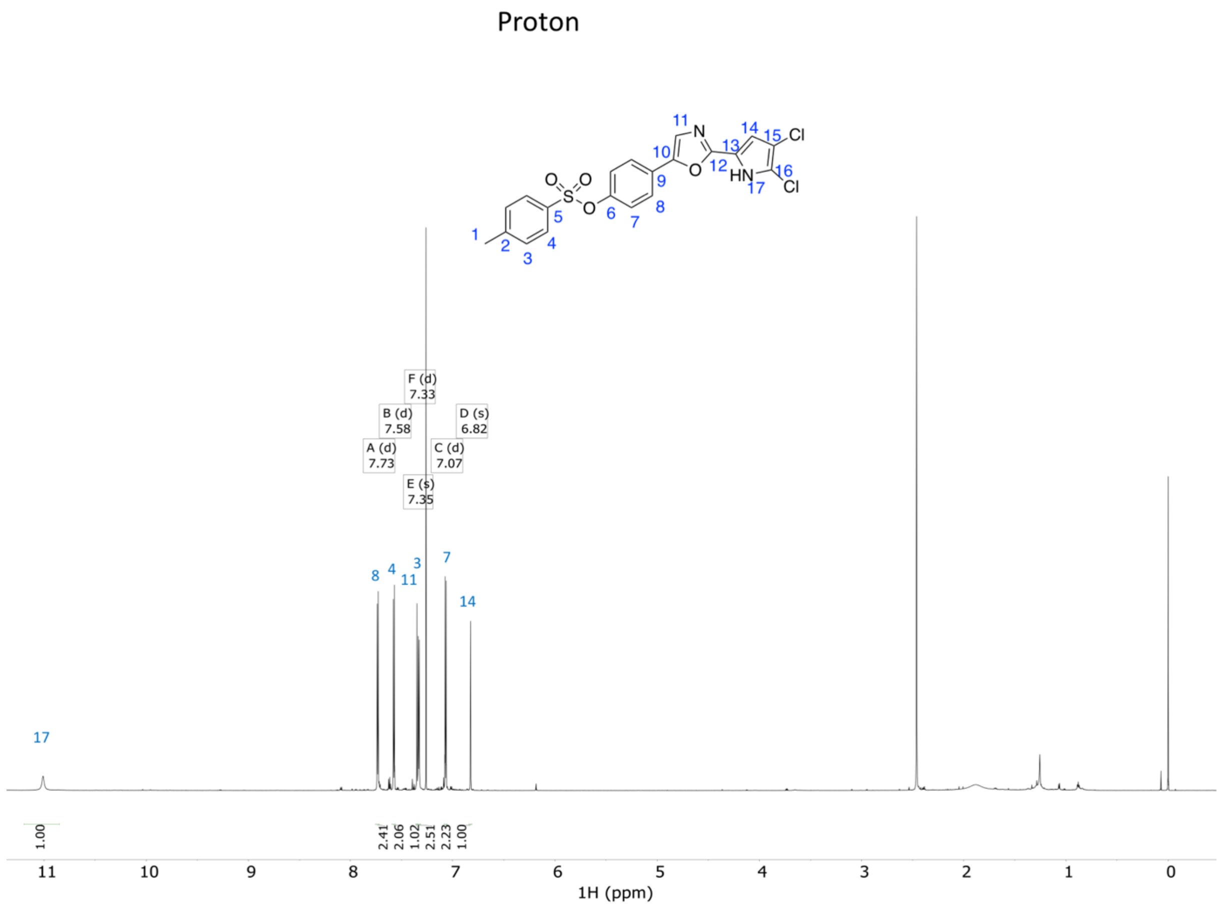

Figure S42. ^{1}H-NMR (850 MHz) spectrum of **24**.

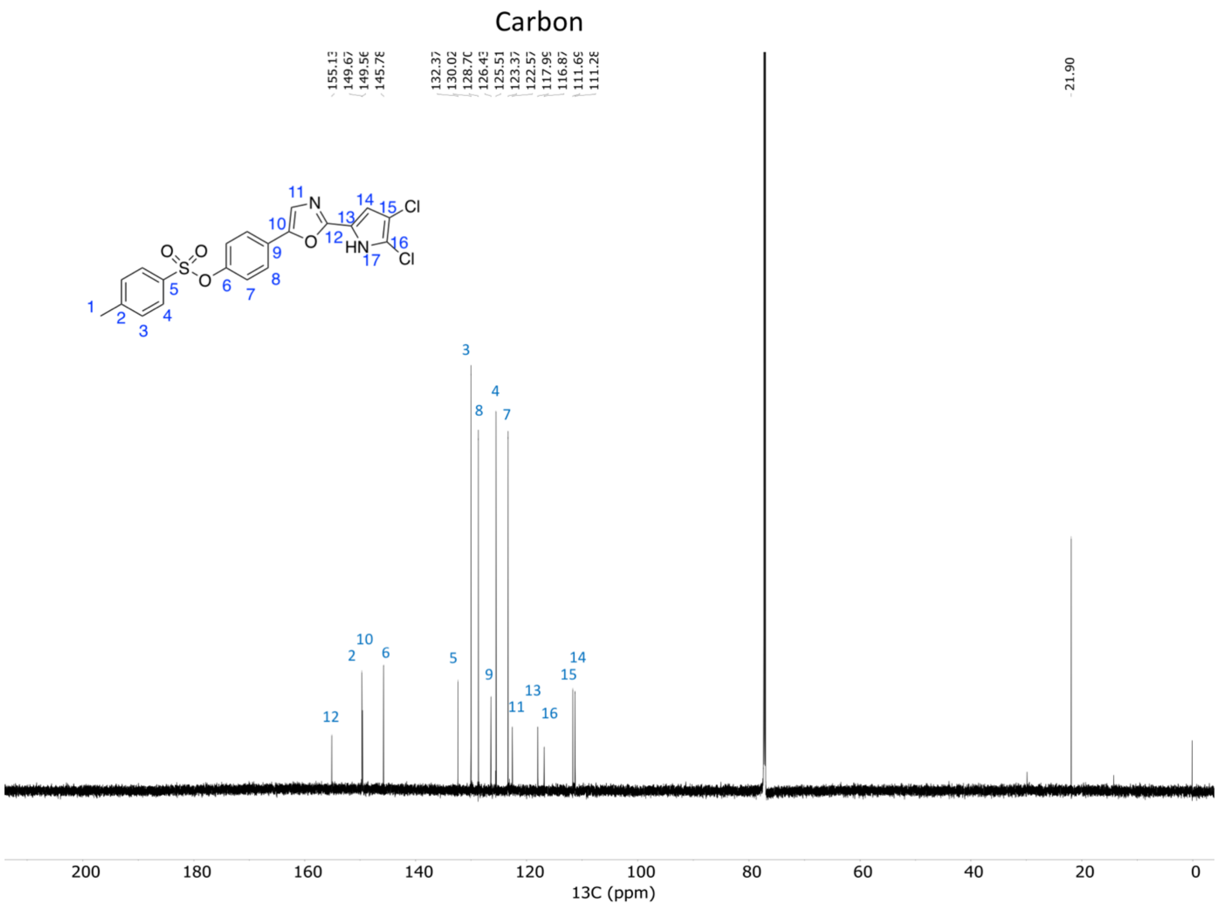

Figure S43. ^{13}C-NMR (214 MHz) spectrum of **24**.

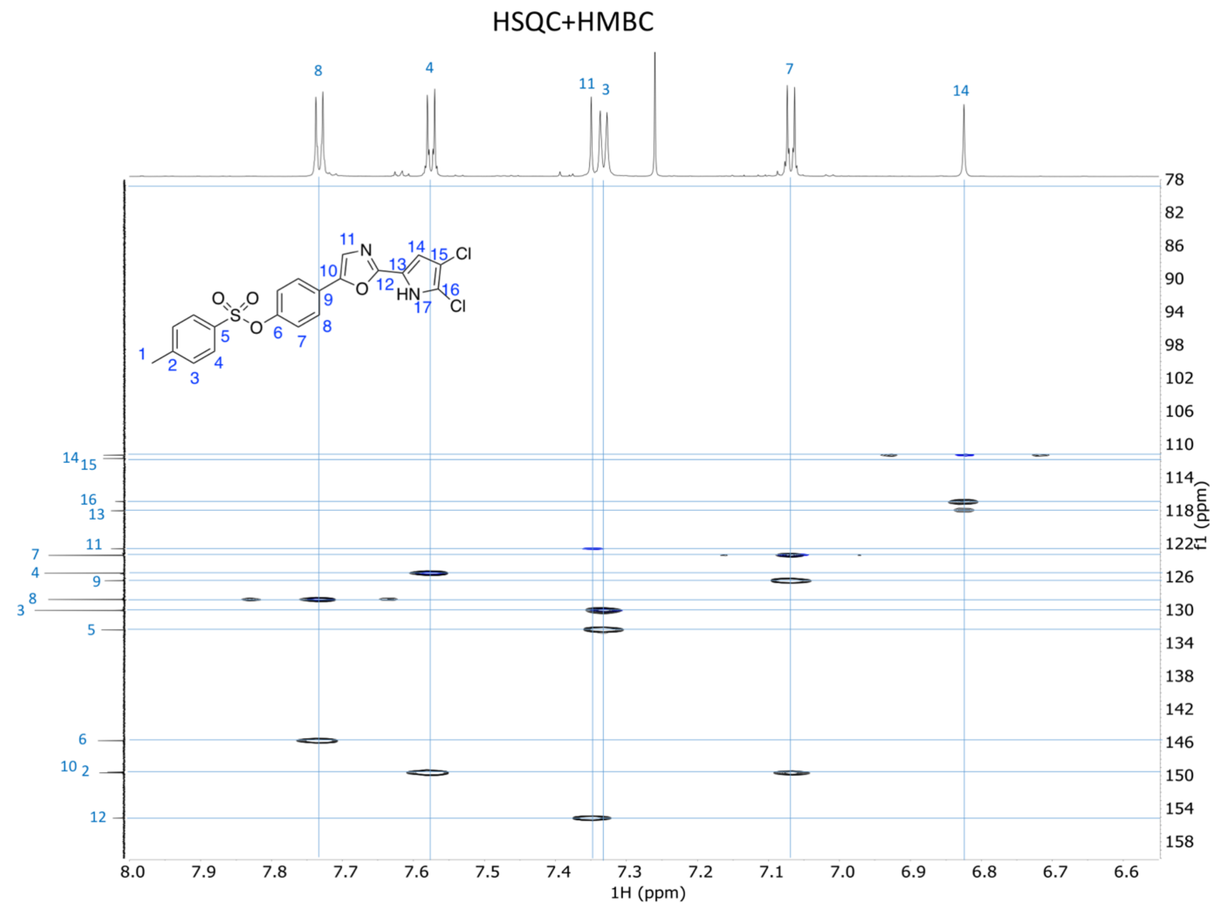

Figure S44. Superimposed HSQC and HMBC of **24**.

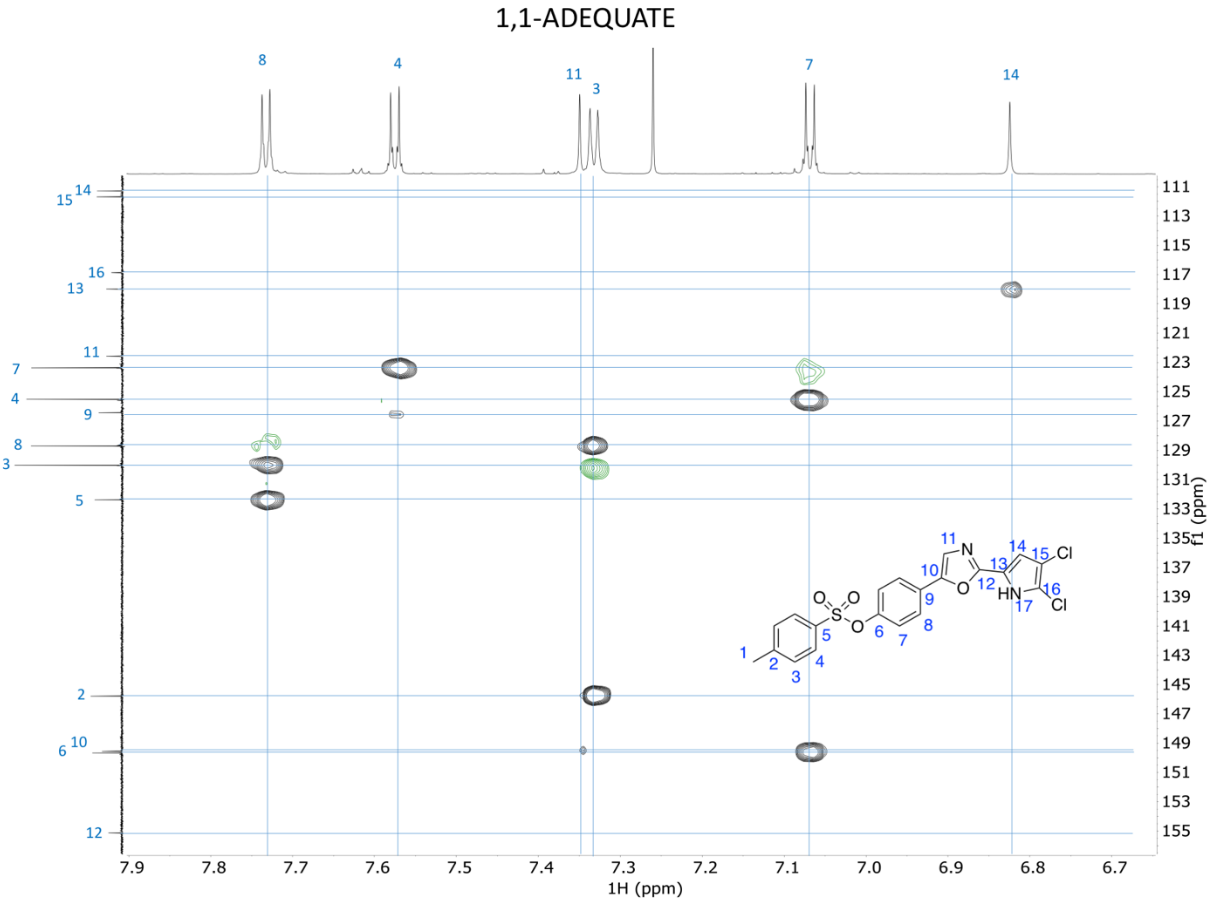

Figure S45. 1,1-ADEQUATE of **24**.

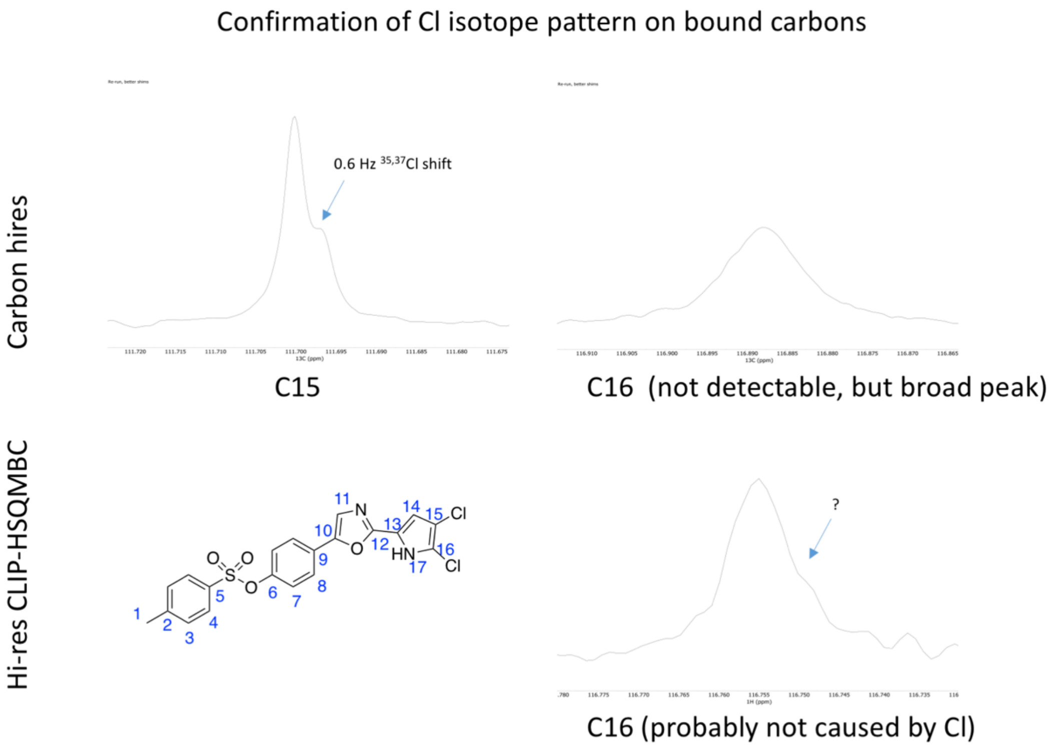

Figure S46. Hires ^{13}C (top panel) and hires selective CLIP-HSQMBC f1 (bottom panel) projection of **24**.

Selective CLIP-HSQMBC

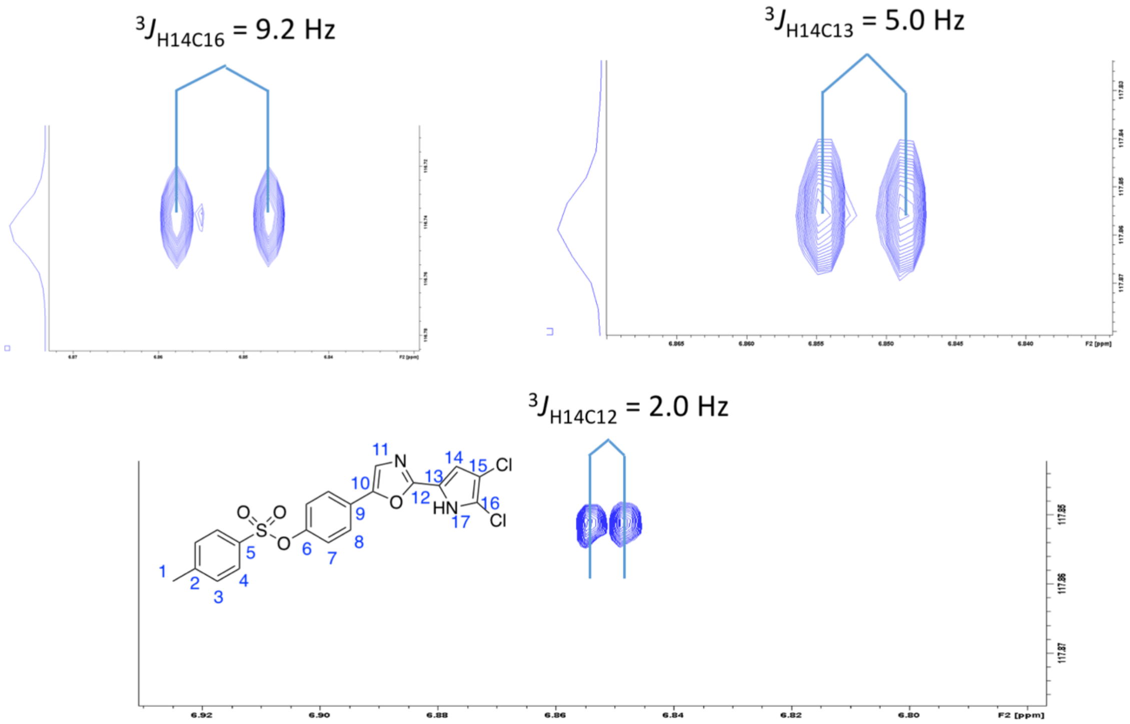

Figure S47. Selective CLIP-HSQMBC of **24**.

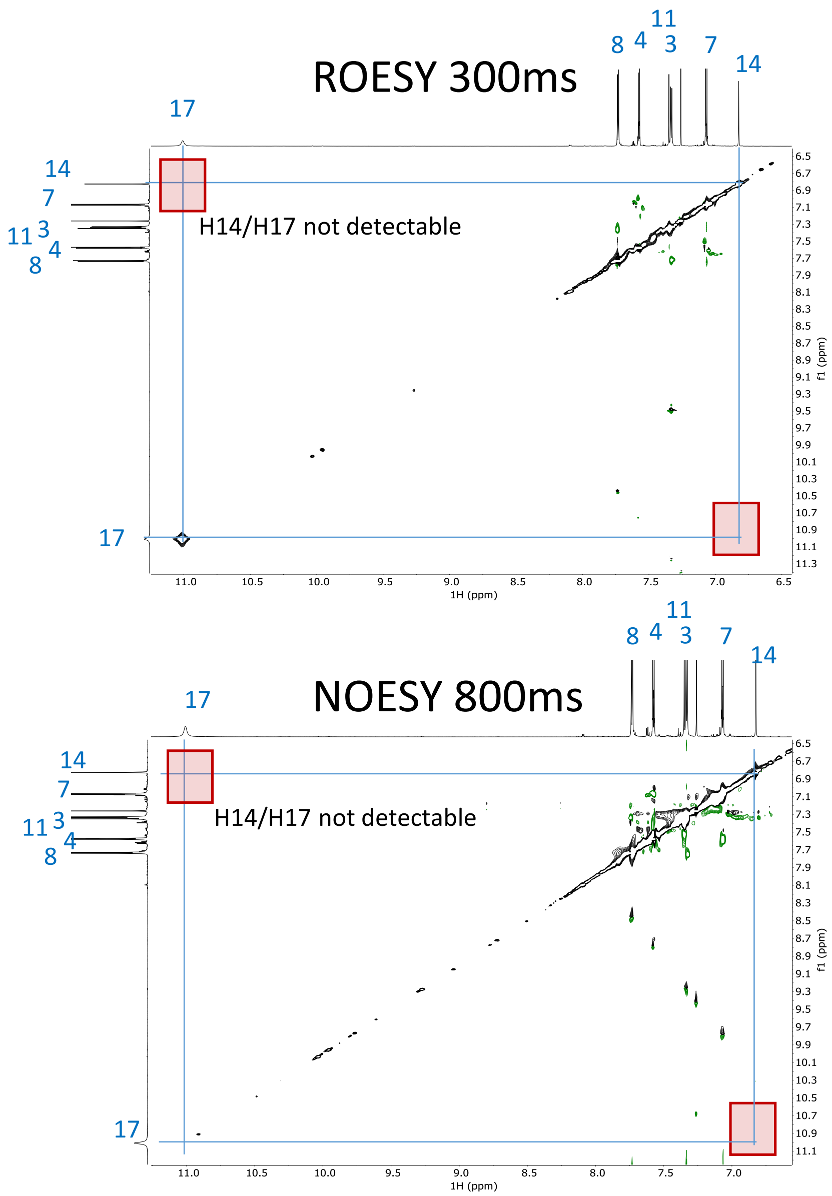

Figure S48. NOESY and ROESY of **24**.

References

1. J. Sauri, M. Reibarkh, T. Zhang, R. D. Cohen, X. Wang, T. F. Molinski, G. E. Martin and R. T. Williamson, *Org. Lett.*, 2016, **18**, 4786-4789.
2. J. Sauri, T. Parella and J. F. Espinosa, *Org. Biomol. Chem.*, 2013, **11**, 4473-4478.
